# Variograms for kriging and clustering of spatial functional data with phase variation

**DOI:** 10.1016/j.spasta.2022.100687

**Published:** 2022-07-10

**Authors:** Xiaohan Guo, Sebastian Kurtek, Karthik Bharath

**Affiliations:** a Department of Statistics, The Ohio State University, 1958 Neil Avenue, Columbus, OH 43210, USA; b School of Mathematical Sciences, University of Nottingham, University Park, Nottingham NG7 2RD, UK

**Keywords:** Spatial amplitude-phase separation, Alignment, Spatial template, Trace-variogram

## Abstract

Spatial, amplitude and phase variations in spatial functional data are confounded. Conclusions from the popular functional trace-variogram, which quantifies spatial variation, can be misleading when analyzing misaligned functional data with phase variation. To remedy this, we describe a framework that extends amplitude-phase separation methods in functional data to the spatial setting, with a view towards performing clustering and spatial prediction. We propose a decomposition of the trace-variogram into amplitude and phase components, and quantify how spatial correlations between functional observations manifest in their respective amplitude and phase. This enables us to generate separate amplitude and phase clustering methods for spatial functional data, and develop a novel spatial functional interpolant at unobserved locations based on combining separate amplitude and phase predictions. Through simulations and real data analyses, we demonstrate advantages of our approach when compared to standard ones that ignore phase variation, through more accurate predictions and more interpretable clustering results.

## 1. Introduction

### 1.1. Motivation

In many disciplines, including environmental science, medicine, biology, geology and econometrics, it is increasingly common to observe functional data with complex spatial dependencies; such data are commonly referred to as spatial functional data (Delicado et al., 2010). An archetypal example is the well-known Canadian weather data consisting of daily temperature recordings at 35 locations across Canada, described in detail in Ramsay and Silverman (2005). Data representing spatial functional data come in the form of traditional spatio-temporal data (Cressie and Wikle, 2011). However, the functional data analysis framework allows one to directly capture temporal variation through its representation, thus enabling one to view data as discrete space–time realizations of a latent functional random field.

From this perspective, spatial functional data analysis can be regarded as an extension of spatial statistical methods to functional data objects. While standard multivariate spatial statistics can be used once some form of dimension reduction of functional data has been carried out (Nerini et al., 2010), the more popular approaches to model spatial correlations directly on observed functions have been based on the notion of a metric-based *trace-variogram*, which extends the standard variogram used in spatial statistics to the setting of second-order stationary, isotropic functional random fields assuming values in the Hilbert space of square-integrable functions (Giraldo et al., 2011). Accordingly, the standard *L*^2^ metric is used in the definition of the trace-variogram which, when coupled with the spatial distance, captures spatial dependencies between functions (Goulard and Voltz, 1993). Specifically, if {*f*_*s*_, *s* ∈ 𝒟} ⊆ *L*^2^ is a second-order stationary and isotropic random field, on a spatial domain 𝒟 with metric *d*, the *L*^2^ trace-variogram function

(1) $$d(r,s)\mapsto V(d(r,s))=\frac{1}{2}E\left({\left\|f_{r}-f_{s}\right\|}^{2}\right),$$

quantifies spatial correlation between functions (∥ · ∥ is the usual *L*^2^ norm). The trace-variogram plays a central role in clustering and kriging of spatially correlated functional data (Mateu and Romano, 2017). For example, coefficients of linear combinations of observed functions that define a linear kriging estimate at a new location are determined using an estimate of the trace-variogram (Giraldo et al., 2011).

A key assumption, implicit with the use of the *L*^2^ distance in the trace-variogram in (1), is that the temporal correspondence between functional observations is fixed. Thus, application of currently available *L*^2^ metric-based trace-variogram methods to spatial functional data either assumes that the functions are perfectly aligned or treats phase variation as negligible noise. In reality, however, as with traditional functional data, it is frequently the case that the observed functions are out of phase: there is temporal misalignment of prominent geometric features of the functions, e.g., local extrema. For example, in the well-studied Canadian temperature dataset, this issue can arise when comparing average daily temperatures for two nearby cities, where in addition to spatial dependency of seasonal high and low temperatures, temporal seasonal trends shared between them should also be considered. Further, underlying phase variation in spatial functional data may easily make it non-stationary.

**Fig. 1.**
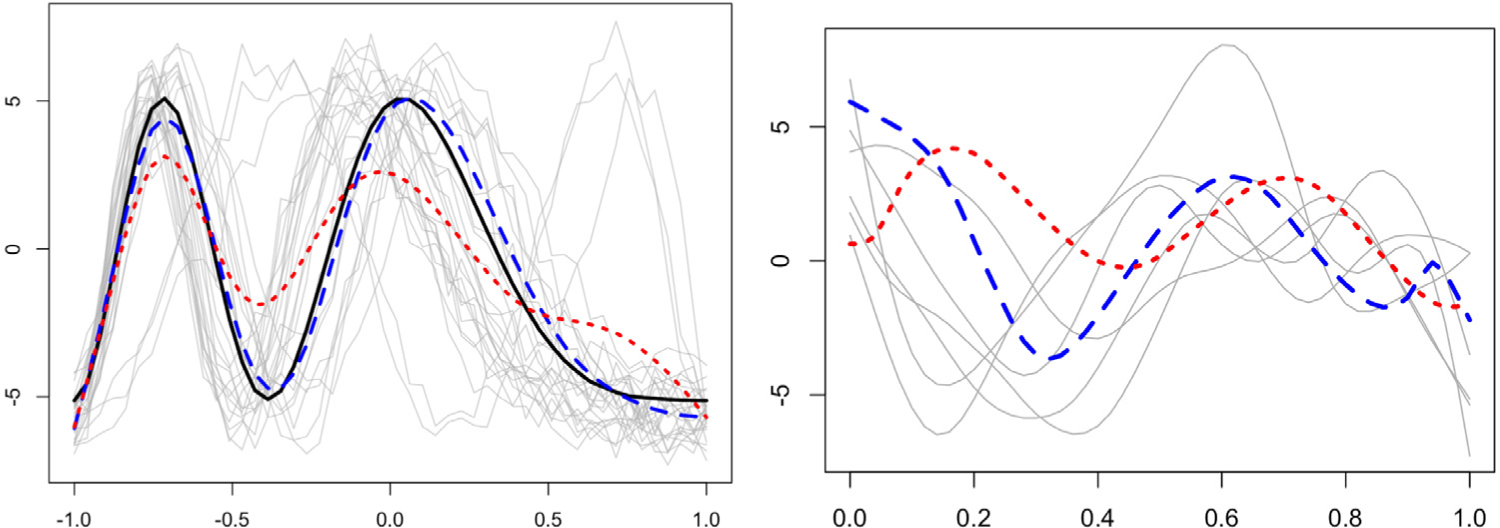
Left: Spatial prediction of a target function (black) based on a sample of spatial functional data (gray) using kriging methods that account for (blue) and ignore (red) phase variation. Right: The local template estimated using spatial correlation (blue) better represents sample variability in a local area (gray) when compared to the (overall) mean amplitude function (red), and is a better template to use for local alignment of functions.

The adverse effects of disregarding phase variation while computing amplitude-related statistical summaries of functional data (e.g., functional mean and functional principal component analysis) using the *L*^2^ distance are well-documented (Marron et al., 2015; Srivastava et al., 2011). The situation is exacerbated in the spatial setting since there are three sources of variation that are potentially confounded: amplitude, phase and spatial, and these have to be appropriately accounted for in the data analysis. A simulated example of kriging of spatial functional data with phase variation is shown in the left panel of Fig. 1. It is clear that the prediction generated by a method that accounts for phase variation (blue) is more accurate than one generated by a method that does not account for phase variation (red). To elaborate, for spatial functional data with phase variation, one is interested in quantifying spatial correlation between two complementary, latent features of the data: amplitude and phase. This necessitates a decomposition of the functional random field {*f*_*s*_, *s* ∈ 𝒟} into its phase {*γ*_*s*_, *s* ∈ 𝒟} and amplitude {*f*_*s*_ ◦ *γ*_*s*_, *s* ∈ 𝒟} components, which should then be used to define appropriate trace-variograms; the phase random field assumes values in the space of warping functions, made precise later, and ◦ denotes function composition. In other words, quantifying spatial variability using a trace-variogram *V* in the presence of phase variation requires a decomposition of *V* into separate amplitude and phase components that are trace-variograms themselves. Such a decomposition will enable more interpretable clustering relating to amplitude and phase components, and will result in better prediction of functions at unobserved locations. This constitutes the main focus of the paper, which to our knowledge has hitherto not been considered.

### 1.2. Contributions

The key challenge in decomposing the traditional trace-variogram *V* into separate amplitude and phase trace-variograms, say, *V*_*a*_ and *V*_*p*_, lies in synthesizing spatial information with the fundamental asymmetry between the absolute and relative notions of amplitude and phase of a function: amplitude variation of a function f:[a,b]→ℝ can be viewed as variability in the set {*f* (*t*)} of *y*-axis values as *t* varies in [*a*, *b*], while phase variation tracks variability in locations along the *x*-axis of amplitude features of *f relative* to another function *g*. As a consequence, any definition of a trace-variogram for phase, based on the variance of the increment $f_{s_{i}-}f_{s_{j}}$ at locations *s*_*i*_, *s*_*j*_ ∈ 𝒟, will depend on the amplitudes (shapes) of the two functions and hence needs to be defined by conditioning on amplitudes (shapes).

Following viable definitions, estimation of *V*_*a*_ and *V*_*p*_ based on a sample of *n* observed functions at locations *s*_1_, …, *s*_*n*_ requires a template function to estimate the unobserved warping functions, by aligning the sample functions to the template. In the absence of spatial correlation, a template is typically estimated by the mean amplitude function (Srivastava et al., 2011). However, when sample functions are spatially correlated, a locally defined (with respect to the spatial domain 𝒟), data-driven template is desirable to better reflect the confounding between, and eventual disentangling of, amplitude, phase and spatial variations. The right panel in Fig. 1 illustrates the advantage of estimating the template using spatial information (blue) over the mean amplitude function (red); the spatial template better reflects amplitude features of the functions observed in a local area (gray). Such a desideratum is particularly relevant for kriging at a new location *s*_0_ at which no functional datum is observed. Operationally, one could first align spatial functional data using any off-the-shelf registration algorithm to separate the amplitude and phase components, followed by appropriate modeling of spatial dependency. But, such an approach does not use local spatial information in the alignment procedure leading to poor results since spatial dependency amongst functions may arise in the amplitude component, the phase component or both; see Appendix A in the supplement for more on this issue. Accordingly, our contributions are as follows.

1. Aided by a geometric framework for amplitude-phase separation in spatial functional data we define separate amplitude and phase trace-variograms (Section 3.1); the amplitude trace-variogram is invariant to warping and hence captures pure amplitude variation (Lemma 1).
2. We propose an algorithm based on a non-trivial extension of the elastic functional data analysis framework (Srivastava and Klassen, 2016) to compute a spatially-weighted template (Algorithm 1) that enables simultaneous alignment and computation of estimators of the amplitude and phase trace-variograms (Section 3.2).
3. Using the trace-variograms, we propose: (i) linear unbiased estimators for kriging of amplitude and phase, which are combined to form the final kriging estimate, and discuss their properties (Sections 4.1–4.3); and (ii) a method for clustering spatial functional data into amplitude and phase clusters (Section 5).

### 1.3. Related work and article organization

Spatial functional data analysis has received considerable attention. Adaptation of multivariate spatial data methods to functional clustering, following dimension reduction, was done in Giraldo et al. (2012) and Haggarty et al. (2015). Romano et al. (2010) and Romano et al. (2017) extended the classical dynamic clustering approach in geostatistics to spatial functional data by employing the trace-variogram. On the other hand, Secchi et al. (2013) introduced Bagging Voronoi classifiers for clustering spatial functional data. This method was further improved by Abramowicz et al. (2017) by combining it with *k*-means registration (Sangalli et al., 2010).

Kriging is based on borrowing information from nearby objects to construct predictions at new spatial locations; the contribution to the predictor from each function depends on the strength of spatial correlation. Giraldo et al. (2011) used the trace-variogram for ordinary kriging of functional observations, which inspired related approaches. Chief amongst these are universal kriging methods wherein observed functions are pre-processed to better manage deviations from the stationarity assumption (Caballero et al., 2013; Menafoglio et al., 2013; Reyes et al., 2015; Menafoglio and Petris, 2016). However, non-stationarity induced by phase variation has not been considered in previous work, and this form of non-stationarity cannot be remedied using the state-of-the-art universal kriging approach (Menafoglio et al., 2013); see simulation results in Section 6.1. Menafoglio et al. (2021) further generalized kriging of functional data to data on a Riemannian manifold.

Indeed, not all functional kriging methods rely on the trace-variogram. Martínez-Hernádez and Genton (2020) outlined a comprehensive list of functional kriging methods. Many of the approaches that do not use the trace-variogram focus on prediction via various forms of penalized regression. Aguilera-Morillo et al. (2017) proposed a functional spatial regression model with penalties accounting for spatial and temporal dependency. Bernardi et al. (2017) proposed a regression approach with partial regularization, and used two roughness penalties that separately accounted for spatial and temporal regularity. Compared to trace-variogram-based approaches, the proposed regression models do not explicitly model spatial dependency of the observations, and ensure regularity of the predictions through penalization.

The rest of this paper is organized as follows. Section 2 introduces the notions of amplitude and phase used throughout this paper, defines amplitude and phase distances used in the specification of amplitude and phase trace-variograms, and discusses template-based alignment to separate amplitude and phase variations. Section 3.1 introduces the proposed amplitude and (conditional) phase trace-variograms while Section 3.2 defines their estimators. Section 4 outlines the procedure for amplitude-phase kriging; a key step is the estimation of a spatially-weighted amplitude template (Algorithm 1). Section 5 introduces amplitude-phase hierarchical clustering based on spatially-weighted dissimilarity matrices. Section 6 reports results of extensive simulations while Section 7 considers applications of the proposed methods on two different datasets. Finally, Section 8 offers a brief discussion and outlines directions for future work. The supplement contains empirical performance assessments of Algorithm 1 (Appendix A), proofs of all propositions (Appendix B), a conceptual model-based formulation for kriging and a discussion of convergence for the proposed amplitude kriging estimator (Appendix C), a discussion of invariance of amplitude-phase clustering to the global scale of the amplitude and (conditional) phase trace-variograms (Appendix D), and additional implementation details and kriging/clustering results (Appendix E).

## 2. Amplitude-phase separation

### 2.1. Relevant function spaces and distances

We build on the metric-based elastic functional data analysis framework for amplitude-phase separation (Srivastava et al., 2011; Srivastava and Klassen, 2016). Without loss of generality, we consider the representation space of functional data objects to be ℱ = {*f* : [0, 1] → *R* | *f* is absolutely continuous}. The group of warping functions representing phase is *Γ* = {*γ* : [0, 1] → [0, 1] | *γ* (0) = 0, *γ* (1) = 1, $\dot{\gamma }>0$} ($\dot{\gamma }$ is the time derivative of *γ*). For any *f* ∈ ℱ, *γ* ∈ *Γ*, the warping of *f* by *γ* is given by the group action of composition, *f* ◦ *γ*. The group-theoretic formulation of phase enables a definition of the amplitude of a function *f* as the equivalence class [*f*] = {*f* ◦ *γ* | *γ* ∈ *Γ*} ⊆ ℱ, known as its orbit under the action of *Γ*; thus, *f* ◦ *γ* ∈ [*f*] has the same amplitude as *f* for each *γ* ∈ *Γ*. The amplitude space then is the quotient space ℱ/*Γ* = {[*f*] | *f* ∈ ℱ}.

Separating amplitude and phase requires a metric on the amplitude space ℱ/*Γ*. A convenient way to define one is through a metric *d* on ℱ that is invariant to simultaneous warping: for every *γ* ∈ *Γ*, *d*(*f*_1_, *f*_2_) = *d*(*f*_1_ ◦ *γ*, *f*_2_ ◦ *γ*). Under such a metric *d*, it becomes possible to view the action of the group *Γ* as performing an isometric operation *γ* ↦ *f* ◦ *γ*, much like an orthogonal transformation *O* ↦ *Ox* for orthogonal matrices *O* and $x\in {\mathbb{R}}^{d}$ that preserves sums of squares of relevant quantities in the multivariate setting.

The standard *L*^2^ metric fails to be invariant and Srivastava et al. (2011) thus proposed to use the extended Fisher–Rao (eFR) metric. Unfortunately, this metric is difficult to use in practice. However, the *square-root slope transform* remarkably reduces the complicated eFR metric on ℱ to the standard *L*^2^ metric on the transformed space. The transform maps $f\mapsto Q(f)=q=\text{sgn}(\dot{f})|\dot{f}{|}^{1/2}$ ($\dot{f}$ is the time derivative of *f*). Given *f* (0), *Q* is bijective with inverse $Q^{-1}(q,f(0))(t)=f(t)=f(0)+\int_{0}^{t}q(u)\left|q(u)\right|du$. Henceforth, for any *f* ∈ ℱ, we will refer to *q* = *Q*(*f*) as its square-root slope function (SRSF).

The transformed space *Q*(ℱ) is a subset of *L*^2^ [0, 1], and, by an abuse of notation, is denoted by 𝒬. Under *Q*, the eFR metric on ℱ maps to the standard *L*^2^ metric on 𝒬, and thus analysis of SRSFs can be carried out using standard Hilbert space machinery. Warping of *f* ∈ ℱ by *γ* induces the warping action $(q,\gamma )=(q\circ \gamma ){\dot{\gamma }}^{1/2}$ on 𝒬 equipped with the *L*^2^ metric, and the action is by isometries since ∥(*q*, *γ*)∥ = ∥*q*∥ for every *γ* ∈ *Γ*, *q* ∈ 𝒬.

The corresponding orbit or *amplitude* of the SRSF *q* is then given by [*q*] = {(*q*, *γ*) | *γ* ∈ *Γ*}, and the *amplitude space* becomes 𝒬/*Γ* = {[*q*] | *q* ∈ 𝒬}. According to this definition, the amplitude of *q* is an entire equivalence class under the action of *Γ*; this implies that each member (*q*, *γ*) of [*q*], as *γ* varies in *Γ*, represents the amplitude component of the function *q*. We will use ‘amplitude’ to refer to both [*q*] and (*q*, *γ*), for any particular *γ*, and the context will disambiguate the two. Note that the amplitude of a function contains its magnitude (global scale), whereas a sensible notion of ‘shape’ of a function would be one that is scale-invariant. We thus define the *shape* of a function as the SRSF orbit $[\bar{q}]=\left\{(\bar{q},\gamma )|\gamma \in \Gamma \right\}$, where $\bar{q}=q/\|q\|$ corresponds to the scale-normalized function, and the set of shapes of functions in 𝒬 constitute the shape space.

**Definition 1** (*Amplitude and Shape Distance*). The amplitude distance between *q*_1_, *q*_2_ ∈ 𝒬 is defined as *d*_*a*_(*q*_1_, *q*_2_) = inf_*γ*∈*Γ*_ ∥*q*_1_ − (*q*_2_, *γ*)∥, where ∥ · ∥ is the *L*^2^ norm, and is a distance on the amplitude space. The shape distance between *q*_1_, *q*_2_ ∈ 𝒬 is defined as $d_{sh}\left(q_{1},q_{2}\right)=d_{a}\left({\bar{q}}_{1},{\bar{q}}_{2}\right)=d_{a}\left(q_{1}/\left\|q_{1}\right\|,q_{2}/\left\|q_{2}\right\|\right)$ and is a distance on the shape space.

Amplitude and phase separation through pairwise registration or alignment of *f*_2_ to *f*_1_ (or vice versa) is formulated as the determination of the relative phase obtained by solving

(2) $$\gamma^{*}=\underset{\gamma \in \Gamma }{\text{arg min}}\left\|q_{1}-\left(q_{2},\gamma \right)\right\|=\underset{\gamma \in \Gamma }{\text{arg min}}\left\|{\bar{q}}_{1}-\left({\bar{q}}_{2},\gamma \right)\right\|,$$

typically using the dynamic programming algorithm, where *q*_1_ and *q*_2_ are the SRSFs of *f*_1_ and *f*_2_, respectively. The optimal alignment of *f*_2_ with respect to *f*_1_ is then given by *f*_2_ ◦ γ*.

Alignment of *f*_2_ to *f*_1_ using *q*_1_ and *q*_2_ allows us to compute their relative phase distance. For this, we consider the square-root slope transform *ψ* of $\gamma :\gamma \mapsto Q(\gamma )=\psi ={\dot{\gamma }}^{1/2}$. Since $\int_{0}^{1}\psi^{2}(t)dt=1$ and *ψ*(*t*) > 0 ∀*t*, the square-root transformed warping group *Q*(*Γ*) = *Ψ* is the positive orthant of the unit sphere in *L*^2^ [0, 1], enabling us to define the (extrinsic) relative phase distance.

**Definition 2** (*Phase Distance*). If $\psi^{*}=\sqrt{{\dot{\gamma }}^{*}}$ is the relative phase between *q*_1_, *q*_2_ ∈ 𝒬, then their (extrinsic) phase distance is *d*_*p*_(*q*_1_, *q*_2_) = ∥ψ* − *ψ*_*id*_∥, where *ψ*_*id*_(*t*) = 1 is the square-root slope transformed identity warping function *γ*_*id*_(*t*) = *t*.

Since *Ψ* is a subset of the unit sphere in *L*^2^ [0, 1], the intrinsic ‘arc-length’ distance cos^−1^(〈ψ*, *ψ*_*id*_〉) can also be used. We note that *Ψ*, equipped with the *L*^2^ Riemannian metric, is a Riemannian manifold. Further, the *L*^2^ metric on *Ψ* corresponds to the Fisher–Rao metric on the warping group *Γ* (Srivastava et al., 2011).

Due to the nonlinear nature of warping, the *L*^2^ distance between *q*_1_, *q*_2_ ∈ 𝒬 does not decompose exactly into the respective amplitude and phase distances in Definitions 1 and 2. The elastic framework, however, enables us to extract pure amplitude and phase components, and disentangle them from spatial variation in spatial functional data.

### 2.2. Template-based alignment of multiple functions

Amplitude-phase decomposition of variability present in a sample *f*_1_, …, *f*_*n*_ can be carried out using the corresponding SRSFs *q*_1_, …, *q*_*n*_ ∈ 𝒬 (equipped with the *L*^2^ metric) by jointly aligning the sample to a template *μ*_*q*_ ∈ 𝒬, which is representative of a population-level amplitude. A natural choice is a representative $\hat{q}$ from the amplitude Karcher mean of [*q*_1_], …, [*q*_*n*_], which is defined as a local minimizer of the variance functional $[q]\mapsto \sum_{i=1}^{n}d_{a}^{2}\left(q,q_{i}\right)$ on the amplitude space 𝒬*/Γ*. In practice, this is carried out by using an algorithm that iterates between aligning {*q*_*i*_} to the current iterate of the representative of the Karcher mean amplitude and updating it (Section 8.3.3 of Srivastava and Klassen, 2016). The output of such an algorithm is the representative $\hat{q}$ and optimal warping functions $\left\{{\hat{\gamma }}_{i}\right\}$, such that (*q*_*i*_, ${\hat{\gamma }}_{i}$) are optimally aligned to $\hat{q}$ with respect to the metric *d*_*a*_. When {*q*_*i*_} are spatially correlated across the spatial domain 𝒟, their amplitudes (and hence the relative phases) are dependent on the locations in 𝒟, and using a common template in their alignment might be inappropriate. We propose a modified version of the above algorithm (Algorithm 1) that jointly computes a suitable template for alignment of *q*_*i*_ *and* carries out the alignment.

### 2.3. Setup and notation

We focus on the setting of dense functional data (Wang et al., 2016), wherein a function at each spatial location is assumed to have been observed on a fine partition of [0, 1]. This implies that we are not considering situations wherein some form of function estimation is required that can potentially add another source of variability to amplitude, phase and spatial variations. It is important to first understand the interplay between the variations in this setting before moving to the more challenging one of sparsely observed functional data.

The functional random field {*f*_*s*_*, s* ∈ 𝒟}, on a spatial domain 𝒟 ⊆ *R*^2^, assumes values in ℱ. Associated with {*f*_*s*_} is its square-root slope transformed version {*q*_*s*_, *s* ∈ 𝒟} such that *s* ↦ *q*_*s*_ ∈ 𝒬. Then, {*q*_*s*_} is a square-integrable functional random field since 𝒬 ⊆ *L*^2^([0, 1]).

Observed functional data $f_{s_{i}},s_{i}\in 𝒟(i=1,\ldots ,n)$ is first mapped to its corresponding SRSF representation, $q_{s_{i}}$, and methodology is entirely developed using $q_{s_{i}}$. Henceforth, the subscript *i* as an index is short for the spatial location *s*_*i*_ (e.g., *q*_*i*_, *γ*_*i*_); the subscript *s* is only used with a functional random field (e.g., *q*_*s*_). The *L*^2^ norm (inner product) on the function spaces 𝒬 and *Ψ* is denoted by ∥ · ∥ (〈·, ·〉), while ∥ · ∥_2_ denotes the Euclidean norm on 𝒟.

## 3. Amplitude-phase separation of trace-variogram

Denote by *μ*_*q*,*s*_ the expected value of the random field {*q*_*s*_} ⊆ 𝒬 defined using the Bochner integral. The covariance function C:𝒟×𝒟→ℝ of {*q*_*s*_} is the positive definite function *C*(*s*, *s*′) = E(〈*q*_*s*_ − *μ*_*q,s*_, *q*_*s*′_ − *μ*_*q,s*′_〉), resulting in the variance function being defined as var(*q*_*s*_) = *C*(*s*, *s*) = E(∥*q*_*s*_ − *μ*_*q,s*_∥^2^). The semi-variogram of the process {*q*_*s*_} then is a conditionally negative definite function defined as $\theta_{q}\left(s,s^{\prime }\right)=\frac{1}{2}\text{var}\left(q_{s}-q_{s^{\prime }}\right)$ for *s*, *s*′ ∈ 𝒟. The random field {*q*_*s*_} is said to be second-order stationary and isotropic if *μ*_*q,s*_ ≡ *μ*_*q*_, i.e., the mean is constant across the spatial domain 𝒟, and *C*(*s*, *s*′) is a function of ∥*s* − *s*′∥_2_ only for every pair (*s*, *s*′). Under this condition, using Fubini’s theorem, the trace-semivariogram *V*_*q*_ corresponds to the integrated pointwise variogram (Giraldo et al., 2011),

(3) $$V_{q}(h)=\theta_{q}(h)=\frac{1}{2}\int_{0}^{1}\text{E}{\left[q_{s}(t)-q_{s^{\prime }}(t)\right]}^{2}\text{d}t=\frac{1}{2}\text{E}\left({\left\|q_{s}-q_{s^{\prime }}\right\|}^{2}\right),$$

where *h* = ∥*s* − *s*′∥_2_. In other words, *V*_*q*_ is (half the) expected squared *L*^2^ distance between values of the functional random field {*q*_*s*_} at two locations in 𝒟. Henceforth, we will simply refer to *V*_*q*_ as the *trace-variogram*. The definition implicitly assumes that *q*_*s*_ and *q*_*s*′_ are aligned with zero phase variation, a situation rarely true in practice. Importantly, *V*_*q*_ is invariant to warping of two SRSF functions *q*_*s*_ and *q*_*s*′_ by the same *γ* ∈ *Γ*. This is not true when the trace-variogram is defined using the *L*^2^ distance on the random field {*f*_*s*_} as in (1), providing a strong motivation for using the SRSF representation to define separate amplitude and phase trace-variograms.

### 3.1. Trace-variograms for amplitude and phase

The amplitude and phase components in spatial functional data represent two distinct sources of variation, and importantly, can have different spatial correlations. Furthermore, in contrast to current approaches, phase variation cannot be viewed as noise. For example, in the aforementioned Canadian weather data (Ramsay and Silverman, 2005), phase represents important seasonal trends of temperature fluctuations across the observed sites, and spatial correlation in the phase component is important to explore regional climate change. Thus, our aim is to define complementary amplitude and phase trace-variograms that separately capture spatial correlation in these two components of spatial functional data, and can be used in downstream statistical tasks.

To define amplitude and phase trace-variograms, we treat the functional random field {*q*_*s*_, *s* ∈ 𝒟} ⊆ 𝒬 as being comprised of two random fields representing amplitude and phase. The amplitude random field is defined as {(*q*_*s*_, *γ*_*s*_), *s* ∈ 𝒟} and the phase random field as {*ψ*_*s*_, *s* ∈ 𝒟}, where *γ*_*s*_ ∈ *Γ* is a random warping function and $\psi_{s}={\dot{\gamma }}_{s}^{1/2}$. Amplitude-phase separation of {*q*_*s*_} into {(*q*_*s*_, *γ*_*s*_)} and {*ψ*_*s*_} allows us to capture the spatial dependence in functional data via two different trace-variograms, one for the amplitude and one for the phase. We provide the definitions of the amplitude and phase trace-variograms next.

**Definition 3** (*Amplitude Trace-Variogram*). Assuming that the amplitude random field {(*q*_*s*_, *γ*_*s*_), *s* ∈ 𝒟} is second-order stationary and isotropic, the amplitude trace-variogram is defined as

(4) $${\left\|s-s^{\prime }\right\|}_{2}=h\mapsto V_{a}(h)=\frac{1}{2}\text{E}\left({\left\|\left(q_{s},\gamma_{s}\right)-\left(q_{s^{\prime }},\gamma_{s^{\prime }}\right)\right\|}^{2}\right).$$

The amplitude trace-variogram is similar to the trace-variogram in (3). The random warping functions *γ*_*s*_ and *γ*_*s*′_ account for the removal of phase variation from the original random field {*q*_*s*_}. Since the amplitude random field is assumed to be stationary, the above definition in essence supposes that any non-stationarity in the functional random field {*q*_*s*_} is induced by phase variation. This is manifestly different from the typical case wherein a spatially dependent mean induces non-stationarity, for which the universal kriging predictor of Menafoglio et al. (2013) may be used. Further, the proposed amplitude trace-variogram is invariant to simultaneous warping of {*q*_*s*_}, which is a direct consequence of the isometric action of *Γ* on 𝒬 under the *L*^2^ metric, as recorded in the following lemma.

**Lemma 1.** *The amplitude trace-variogram in* (4) *is invariant to simultaneous warping of the functional random field* {*q*_*s*_, *s* ∈ 𝒟} *by any γ* ∈ *Γ*.

While it may be reasonable to assume that the amplitude random field {(*q*_*s*_, *γ*_*s*_)} is second-order stationary and isotropic on 𝒟, elements of the phase random field {*ψ*_*s*_} are only relative and generally depend on both (proximity of) spatial locations and similarity in the *shapes of the functions* that constitute the random field {*q*_*s*_}. Thus, to account for the relative nature of phase, it is thus sensible to consider the phase random field {*ψ*_*s*_} conditional on the shape random field associated with {*q*_*s*_} defined as $𝒮=\left\{\left[{\bar{q}}_{s}\right],\;s\in 𝒟\right\}$ (recall that ${\bar{q}}_{s}=q_{s}/\left\|q_{s}\right\|$). This allows us to handle the non-stationarity in the phase random field {*ψ*_*s*_} due to heterogeneous shapes of the functions in the random field {*q*_*s*_}. The relative nature of phase, with respect to amplitude or shape features, has received considerable attention in previous literature, albeit in other statistical contexts. For example, Sangalli et al. (2010) propose a simultaneous approach for clustering and alignment of functional data, where the cluster partitions are determined via amplitude similarity, and the relative phase of each function is estimated with respect to a cluster-specific template. As a result, the procedure accounts for the fact that only functions with similar amplitude have comparable phase components. Strait et al. (2017) and Matuk et al. (2021) further show that using shape constraints to regularize the phase component of functions and/or curves can result in more natural alignment. This leads to the following definition of the conditional phase trace-variogram.

**Definition 4** (*Conditional Phase Trace-Variogram*). Assuming that, conditional on the shape random field 𝒮, the phase random field {*ψ*_*s*_, *s* ∈ 𝒟} is second-order stationary and isotropic, the conditional phase trace-variogram is defined as

(5) $$V_{p}\left({\left\|s-s^{\prime }\right\|}_{2},𝒮\right)=\frac{1}{2}E\left({\left\|\psi_{s}-\psi_{s^{\prime }}\right\|}^{2}|𝒮\right).$$

The above definition requires a valid definition of distance on 𝒟 that uses information of the shape random field 𝒮. Inspired by the approach proposed by Schmidt et al. (2011) for traditional spatial data, we consider shape as an additional covariate in order to define a pseudo-metric on a subset $ℳ≔\{\left(s,\left[{\bar{q}}_{s}\right]\right)|s\in 𝒟$, $\left[{\bar{q}}_{s}\right]\in 𝒮\}\subset 𝒟\times 𝒮$. For a fixed *ω* ∈ *R*_≥0_, define a functional *h*_*ω*_ : ℳ × ℳ → *R*_≥0_ as

(6) $$h_{\omega }=h_{\omega }\left(\left(s,\left[{\bar{q}}_{s}\right]\right),\left(s^{\prime },\left[{\bar{q}}_{s^{\prime }}\right]\right)\right)=\sqrt{{\left\|s-s^{\prime }\right\|}_{2}^{2}+\omega \cdot d_{sh}^{2}\left(q_{s},q_{s^{\prime }}\right)},\omega \geq 0,\;\left(s,\left[{\bar{q}}_{s}\right]\right),\;\left(s^{\prime },\left[{\bar{q}}_{s^{\prime }}\right]\right)\in ℳ,$$

that provides a combined measure of discrepancy between shapes $\left[{\bar{q}}_{s}\right]$ and $\left[{\bar{q}}_{s^{\prime }}\right]$ of two functions at locations *s* and *s*′ and their spatial distance; *ω* serves as a tuning parameter that allows us to adjust the importance of the shape covariate. Thus, we consider a modification of the conditional phase trace-variogram *V*_*p*_ defined as

$$V_{p}\left(h_{\omega }\right)=V_{p}\left({\left\|s-s^{\prime }\right\|}_{2},𝒮\right)=\frac{1}{2}\text{E}\left({\left\|\psi_{s}-\psi_{s^{\prime }}\right\|}^{2}|𝒮\right).$$

Reminiscent of the pseudo-metric E[(*x*_*s*_ − *x*_*s*′_)^2^]^1/2^ on 𝒟 for a Gaussian random field {*x*_*s*_, *s* ∈ 𝒟} (see, e.g., Section 1.3 of Adler and Taylor, 2007), one can view *h*_*ω*_ as a pseudo-distance on the spatial domain 𝒟, and its definition is motivated by the fact that the relative phase components of functions with very different shapes are not comparable. In other words, for a fixed *ω* > 0, when two functions have very different shapes, their phase components are viewed as ‘spatially’ far away from each other in terms of the pseudo-distance *h*_*ω*_. Viewing function shape information as an additional covariate (or coordinate), the relative phase components of {*q*_*s*_*, s* ∈ 𝒟} are further stratified according to the shapes of the associated functions. This idea is analogous to the one used in Sangalli et al. (2010) for simultaneous clustering and alignment of functional data; the main difference lies in the use of a continuous measure of shape discrepancy in our case versus a discrete partition of the function space in theirs. As will be seen in the sequel, the estimator of the proposed conditional phase trace-variogram better captures the interplay between relative phases and spatial dependencies of the sample functions. Henceforth, we refer to the conditional phase trace-variogram simply as the phase trace-variogram.

**Remark 1.** Introducing shape information in the phase trace-variogram allows us to account for the potential association between amplitude and phase components in spatial functional data. As defined, the phase trace-variogram considers a functional random field over an infinite-dimensional domain, i.e., the space ℳ ⊂ 𝒟 × 𝒮. Literature on variography over infinite-dimensional spaces is scarce, and we use the proposal in this paper without formal theoretical justification. That said, we have found through extensive simulations and real data applications that the phase trace-variogram defined in (5) has strong practical value; see Sections 6 and 7, and Appendix E in the supplement. A rigorous examination of the conditional phase trace-variogram is a significant undertaking and beyond the scope of this manuscript; as such, we leave it as future work. Alternatively, one could define the phase trace-variogram by replacing *d*_*sh*_ in (6) with a distance on a space of reduced (finite) dimension that captures shape features of the functions. Dimension reduction in this case can be attained either through functional principal component analysis or an appropriate basis decomposition. However, the choice of dimension reduction procedure will have a strong effect on the resulting distance and phase trace-variogram.

**Fig. 2.**
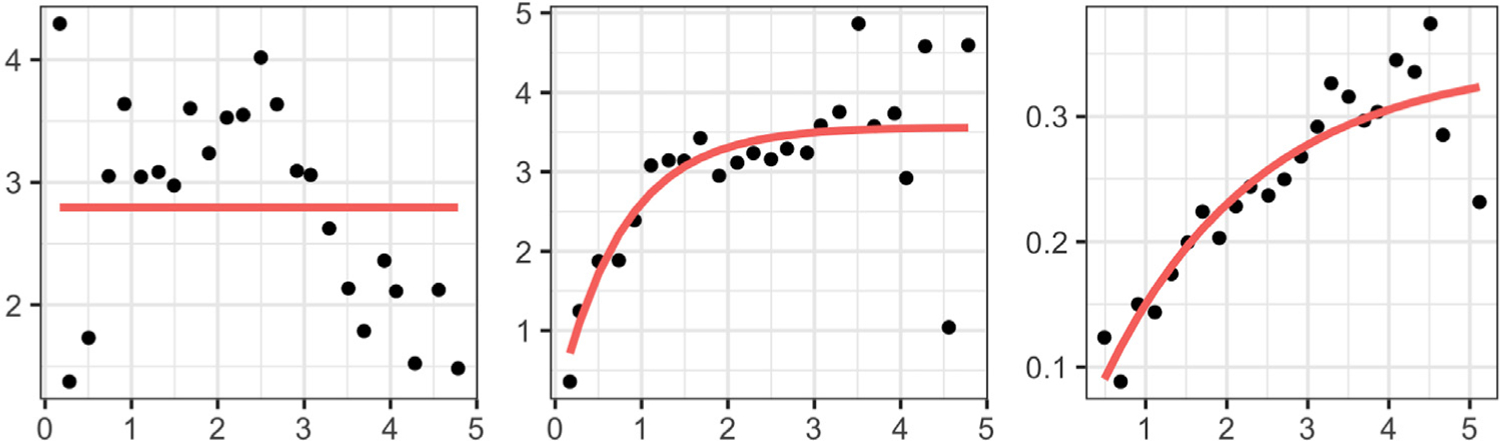
Decomposition of the *L*^2^ trace-variogram (left) into amplitude (middle) and phase (right) components for simulated functional data with spatially correlated amplitudes and phases. The dots represent the empirical *L*^2^, amplitude, and phase trace-variograms (see definitions in Section 3.2). Estimates of the trace-variograms (red curves) are obtained by fitting a Matérn variogram model to the empirical trace-variograms.

The benefits of constructing separate amplitude and phase trace-variograms are illustrated in Fig. 2 using simulated functions, wherein the spatial dependency in the data arises through both the amplitude and phase components. The amplitude components are generated from a second-order stationary and isotropic functional random field, whereas the correlation between phase components arises through both, their spatial locations and the shape features of the associated functions. Failure to disentangle the amplitude and phase variations leads to an empirical trace-variogram (Delicado et al., 2010) that suggests a quadratic pattern for spatial dependency (left panel), which is the truth for neither amplitude nor phase. A fitted Matérn variogram model, shown in red, is constant and fails to capture the spatial correlation that exists in the data. On the other hand, decomposing the trace-variogram into amplitude and phase (the empirical versions of (4) and (5)) appropriately captures the spatial correlatedness (middle and right panels) in these two components.

### 3.2. Estimating amplitude and phase trace-variograms

We have introduced the definitions of trace-variograms using latent amplitude and phase components. The amplitude and phase of given spatial functional data, however, are not observable and need to be estimated through appropriate alignment procedures that satisfy the requirements of different statistical analysis tasks. Here, given a sample of functions {*q*_*i*_, *s*_*i*_ ∈ 𝒟} (*i* = 1, …, *n*), we propose empirical versions of the amplitude and phase trace-variograms that are compatible with the kriging and clustering tasks.

For kriging at a new location, since information from the entire sample *q*_1_, …, *q*_*n*_ is used, we require a template-based multiple alignment approach. For this, it is essential to define a sensible template that captures spatially localized features of the sample. A detailed algorithm for estimating such a template is given in Section 4. Assuming that a template is available, we extract the relative phase components $\left\{{\hat{\gamma }}_{i}\right\}$ by aligning each function in the sample *q*_1_, …, *q*_*n*_ to the template. The aligned functions {(*q*_*i*_, ${\hat{\gamma }}_{i}$)} and estimated (transformed) warping functions $\left\{{\hat{\psi }}_{i}\right\}$ are then used to estimate the amplitude and phase trace-variograms, respectively. Specifically, the empirical amplitude trace-variogram is

(7) $${\hat{V}}_{a}(h)=\frac{1}{2\left|N_{a}(h)\right|}\sum_{i,j\in N_{a}(h)}{\left\|\left(q_{i},{\hat{\gamma }}_{i}\right)-\left(q_{j},{\hat{\gamma }}_{j}\right)\right\|}^{2},$$

where *N*_*a*_(*h*) = {(*s*_*i*_, *s*_*j*_) | ∥*s*_*i*_ − *s*_*j*_∥ = *h*}. For irregularly spaced data, *N*_*a*_(*h*) is modified to {(*s*_*i*_, *s*_*j*_) : ∥*s*_*i*_ − *s*_*j*_∥ ∈ (*h* − *ϵ*, *h* + *ϵ*)} for a small *ϵ* > 0. Similarly, a feasible estimator of *V*_*p*_ is

(8) $${\hat{V}}_{p}\left(h_{\omega }\right)=\frac{1}{2\left|N_{p}\left(h_{\omega }\right)\right|}\sum_{i,j\in N_{p}\left(h_{\omega }\right)}{\left\|{\hat{\psi }}_{i}-{\hat{\psi }}_{j}\right\|}^{2},$$

where the neighborhood $N_{p}\left(h_{\omega }\right)=\left\{\left(\left(s_{i},\left[{\bar{q}}_{i}\right]\right),\left(s_{j},\left[{\bar{q}}_{j}\right]\right)\right)|h_{\omega }\left(\left(s_{i},\left[{\bar{q}}_{i}\right]\right),\left(s_{j},\left[{\bar{q}}_{j}\right]\right)\right)\in \left(h_{\omega }-\epsilon ,h_{\omega }+\epsilon \right)\right\}$ (for a small *ϵ* > 0) is defined with respect to the pseudo-distance *h*_*ω*_ specified in (6), with a suitable choice of *ω* ≥ 0.

The estimators ${\hat{V}}_{a}$ and ${\hat{V}}_{p}$ are simplified when only pairwise comparisons of functions *q*_1_, …, *q*_*n*_ are of interest; this is the case for example in clustering methods based on dissimilarity/distance matrices such as hierarchical clustering. Here, a joint alignment of *q*_1_, …, *q*_*n*_ to a template can be avoided, with alignment between *q*_*i*_ and *q*_*j*_ carried out using either of the functions as the template. This circumvents the challenges associated with estimation of a template, and thus reduces the computational and methodological complexity. In this case, the corresponding expressions for ${\hat{V}}_{a}$ and ${\hat{V}}_{p}$ reduce to

(9) $${\hat{V}}_{a}(h)=\frac{1}{2\left|N_{a}(h)\right|}\sum_{i,j\in N_{a}(h)}d_{a}{\left(q_{i},q_{j}\right)}^{2},\;{\hat{V}}_{p}\left(h_{\omega }\right)=\frac{1}{2\left|N_{p}\left(h_{\omega }\right)\right|}\sum_{i,j\in N_{p}\left(h_{\omega }\right)}d_{p}{\left(q_{i},q_{j}\right)}^{2},$$

where *N*_*a*_(*h*) and *N*_*p*_(*h*_*ω*_) are defined as in (7) and (8).

**Fig. 3.**
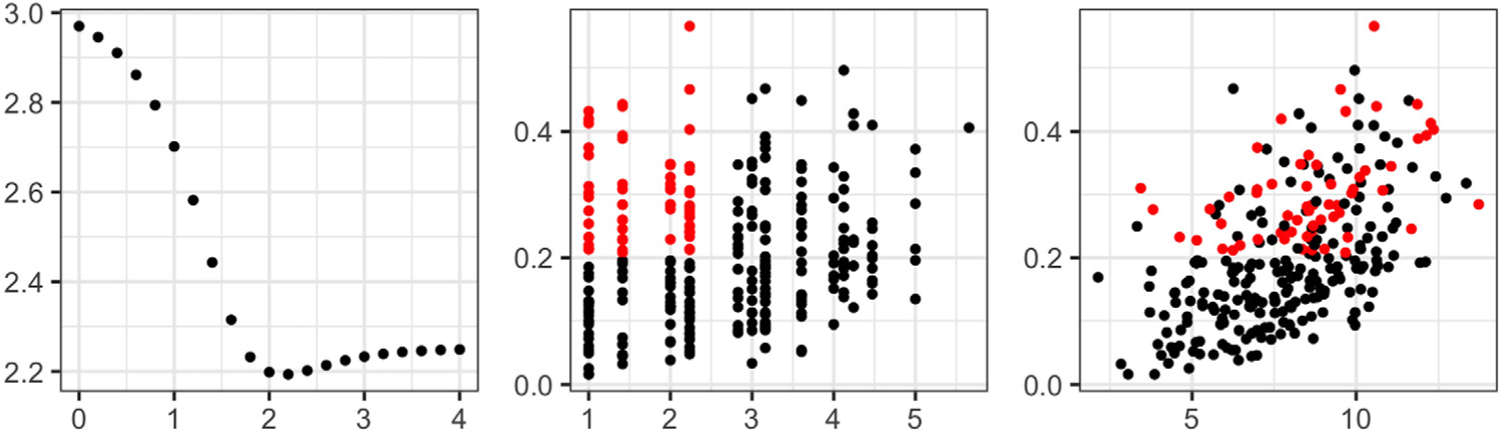
Left: Squared error (*y*-axis) of Matérn variogram fit to the empirical phase trace-variogram (8) under different values of log_10_(*ω*) (*x*-axis). Middle: The pairwise squared distances ${\left\|{\hat{\psi }}_{i}-{\hat{\psi }}_{j}\right\|}^{2}$ (y-axis) versus pairwise pseudo-distances *h*_*ω*_ (*x*-axis) with *ω* = 0. Right: Same as middle for *ω* = 10^2.2^. Red points represent pairs with small pseudo-distance *h*_*ω*_, but relatively large phase discrepancy.

To guarantee that the estimated variograms are conditionally negative definite (Cressie, 2015), we fit a Matérn variogram model to the empirical variograms at a discrete set of distance values; we use ordinary least squares (Cressie, 2015) to estimate the parameters of the Matérn model. In subsequent analyses, i.e., kriging and clustering, the fitted variograms are used instead of the empirical variograms ${\hat{V}}_{a}$ and ${\hat{V}}_{p}$. In the phase trace-variogram, the tuning parameter *ω* is selected to minimize the squared error of the parametric Matérn fit to the empirical estimate. Fig. 3 illustrates an example of tuning parameter selection for a simulated dataset. In the left panel, we plot the squared error (*y*-axis) of the fit versus different values of *ω* (on the log_10_ scale on the *x*-axis). In the middle (*ω* = 0) and right (optimal *ω* = 10^2.2^) panels, spatial correlation patterns of the phase components are captured by scatterplots of the pairwise phase discrepancies ${\left\|{\hat{\psi }}_{i}-{\hat{\psi }}_{j}\right\|}^{2}$ (*y*-axis) versus the pairwise pseudo-distances *h*_*ω*_ (*x*-axis). In red, we highlight points corresponding to small spatial distances (∥*s*_*i*_ − *s*_*j*_∥), but relatively large phase discrepancies. After introducing shape information as a covariate through the pseudo-distance *h*_*ω*_, with *ω* = 10^2.2^, the red points shift to the right due to shape differences of the corresponding functions. This shows that the large phase discrepancy in the red points in the middle panel is partially due to the shape differences of the corresponding functions. In the right panel, using the optimal value of the tuning parameter, we are able to account for the shape heterogeneity in the given data, and as a result, detect a clearer dependency pattern between the phase components. Note that the scale of *ω* in the distance *h*_*ω*_ depends on the difference in the scales of the spatial and shape distances. In particular, one can show that the shape distance *d*_*sh*_ is bounded above by 2. The spatial distance, on the other hand, depends on the size (and coordinates) of the spatial domain. Thus, there is no absolute scale for the tuning parameter *ω*.

## 4. Amplitude-phase kriging

Giraldo et al. (2011) developed a linear unbiased estimator that extends ordinary kriging or spatial interpolation to the functional setting by minimizing the *L*^2^ prediction error. In the presence of phase variation, the *L*^2^-based linear estimator can be biased, since function features such as local extrema can be misaligned; see an example in the left panel of Fig. 1. Given observations {*q*_*i*_, *s*_*i*_ ∈ 𝒟} (*i* = 1, …, *n*), the goal is to predict an unobserved function *q*_0_ at a new location *s*_0_ ∈ 𝒟 comprising amplitude (*q*_0_, *γ*_0_) and phase *γ*_0_. To address possible misalignment of *q*_*i*_, we consider a three-stage kriging procedure: (i) predict the amplitude component, (ii) predict the phase component conditional on the predicted amplitude, and (iii) combine the two to obtain the kriging estimate.

### 4.1. Amplitude kriging

Let us first assume that a template has been chosen so that a multiple alignment procedure has been implemented to obtain aligned functions {(*q*_*i*_, ${\hat{\gamma }}_{i}$)} and warping functions $\left\{{\hat{\gamma }}_{i}\right\}$; for each *i* = 1, …, *n*, recall that (*q*_*i*_, ${\hat{\gamma }}_{i}$) is an estimate of the amplitude [*q*_*i*_] of *q*_*i*_ as a representative of the orbit. Let $\Delta_{n}≔\left\{{\left(x_{1},\ldots ,x_{n}\right)}^{T}\in R^{n}|\sum_{i=1}^{n}x_{i}=1\right\}$. We define the linear estimator of the amplitude component (*q*_0_, *γ*_0_) at *s*_0_ as

(10) $${\tilde{q}}_{0}(t)=\sum_{i=1}^{n}\eta_{i}\left(q_{i},{\hat{\gamma }}_{i}\right)(t),$$

where the coefficient vector *η* = (*η*_1_, …, *η*_*n*_)^*T*^ ∈ Δ_*n*_ is implicitly defined as the minimizer of the expected amplitude prediction error functional

(11) $$\eta \mapsto \text{E}\left({\left\|{\tilde{q}}_{0}-\left(q_{0},\gamma_{0}\right)\right\|}^{2}\right).$$

In the ideal setting, if {*γ*_*i*_} are known or can be estimated exactly, the situation reduces to the traditional setting without phase variation for determining *η* that simplifies the optimization in (11) through its relationship with a matrix consisting of evaluations of the trace-variogram (see, e.g., Menafoglio et al. (2013)). The following result demonstrates this.

**Proposition 1.** *If we assume that γ*_*i*_ *can be estimated exactly such that* (*q*_*i*_, ${\hat{\gamma }}_{i}$) = (*q*_*i*_, *γ*_*i*_) *for every i* = 1, …, *n, then the η* ∈ Δ_*n*_ *that minimizes* (11) *also minimizes η* ↦ *η*^*T*^ 𝒱_*a*_*η, where the n* × *n matrix* 𝒱_*a*_ *contains as its elements V*_*a*_(*h*_0j_) + *V*_*a*_(*h*_i0_) − *V*_*a*_(*h*_*ij*_) *with h*_*ij*_ = ∥*s*_*i*_ − *s*_*j*_∥_2_ (*i, j* = 1, …, *n*). *As a consequence, the amplitude kriging predictor in* (10) *depends only on the amplitude trace-variogram V*_*a*_(*h*).

Unfortunately, it is well-known that the ideal setting considered in Proposition 1, wherein the phase components *γ*_*i*_ can be estimated exactly, only applies in very restrictive modeling scenarios (Kurtek and Srivastava, 2011; Chakraborty and Panaretos, 2021); a more detailed discussion of this issue is given in Appendix C in the supplement. An important implication of this fact is that the estimation of amplitude and phase components heavily relies on the choice of template, and thus it is essential to estimate an appropriate template in the amplitude-phase separation for kriging.

When aligning independent functional data, as described in Section 2.2, the template is typically estimated by the mean amplitude function (a representative of the mean amplitude orbit), i.e., the minimizer of the variance functional. However, when the sample functions are spatially correlated, the dependence structure must be taken into account in template estimation. When focusing on prediction at a certain location *s*_0_, we seek a local (with respect to the spatial domain 𝒟) template that captures the amplitude features of aligned functions that have (strong) spatial correlation with the amplitude component at *s*_0_. In particular, the ideal template for alignment of *q*_1_, …, *q*_*n*_ for amplitude kriging at *s*_0_ is an element of the orbit [*q*_0_]. However, *q*_0_ (and its orbit) is not observed as it is the quantity we seek to predict. This implies that the two objectives of (i) estimating a template for alignment of spatial functional data, and (ii) prediction of amplitude at the location *s*_0_ are essentially the same. We outline a procedure, provided as Algorithm 1, that iterates between the following two steps until convergence: (i) alignment of {*q*_*i*_, *s*_*i*_ ∈ 𝒟} given the current estimate of the template, and (ii) prediction of amplitude at *s*_0_ that specifies an update of the template. The algorithm results in a *spatially-weighted amplitude kriging estimator ${\tilde{q}}_{0}$ that serves the dual purpose of acting as a local template for alignment and as a predictor of the amplitude component* (*q*_0_, *γ*_0_).


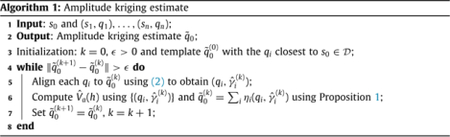


In Algorithm 1, within each iteration *k*, the template ${\tilde{q}}_{0}^{\left(k\right)}$ is fixed, and acts as the given template in Proposition 1. Spatial information is incorporated through the use of ${\hat{V}}_{a}(h)$ for determining *η*. Strictly speaking, the equivalent formulation of the optimization criterion to determine *η* in Proposition 1 assumes that $\left\{{\hat{\gamma }}_{i}\right\}$ recover {*γ*_*i*_} exactly; we nevertheless use it as it significantly simplifies computations. As a result, Algorithm 1 specifies a (spatially) local alignment procedure that emphasizes functions observed at locations close to *s*_0_ based on a local template.

**Remark 2.** Explicit convergence analysis for Algorithm 1 is complicated due to the alternating optimizations to compute the phase functions $\left\{{\hat{\gamma }}_{i}\right\}$, and the weights *η* to construct the spatially-weighted template, at each iteration. In particular, due to the dependence of *η* in line 6 on the phase functions estimated in line 5, it is difficult to formalize the entire procedure under a single cost function. We empirically examine convergence properties of Algorithm 1 in Appendix A in the supplement. Additionally, in Appendix C in the supplement, we establish convergence of the algorithm for a one-dimensional model when randomness manifests in *q* only through a scale parameter.

The advantages of using the local template over a global one (e.g., Karcher mean of amplitude) are confirmed by simulations in Appendix A in the supplement. In particular, it is evidenced there that the local template, estimated via Algorithm 1, better reflects amplitude features of the sample functions observed in a (spatially) local area than a global template that does not take spatial dependence into account. In Appendix A, we also evaluate the influence of the initialization ${\tilde{q}}_{0}^{\left(0\right)}$ in line 3 on prediction performance.

### 4.2. Phase kriging

In amplitude kriging, phase variability is removed by aligning all functions with respect to the estimated template, which results in improved prediction of the shape and magnitude of a function (Section 6). However, ${\tilde{q}}_{0}$ is the prediction of an element in [*q*_0_] and not *q*_0_. To obtain the final kriging estimate of *q*_0_, we require an estimator of the phase *γ*_0_. We construct one by carrying out phase kriging using the estimated warping functions $\left\{{\hat{\gamma }}_{i}\right\}$, with corresponding square-root slope transforms $\left\{{\hat{\psi }}_{i}\right\}$, computed by aligning {*q*_*i*_} to the amplitude kriging estimate ${\tilde{q}}_{0}$.

We want to predict *ψ*_0_ on *Ψ*, which is a nonlinear Riemannian manifold, using the relative phases ${\hat{\psi }}_{1},\ldots ,{\hat{\psi }}_{n}$. We deal with the nonlinearity of *Ψ* by considering the positive extension of *Ψ*, $\Psi^{\prime }=\left\{\tilde{\psi }=a\psi |a\in R_{+},\psi \in \Psi \right\}$, i.e., we embed *Ψ* in *L*^2^ [0, 1] via *Ψ*′. Compatible with the linearity of the amplitude kriging estimate ${\tilde{q}}_{0}$, we use an extrinsic approach to phase kriging: we compute the corresponding linear phase kriging estimate in *Ψ*′ and then project it back to *Ψ*. The projection *Π* : *Ψ*′ → *Ψ* is defined as $\Pi (\tilde{\psi })=\text{arg }{\text{min}}_{\psi \in \psi }\|\psi -\tilde{\psi }\|=\tilde{\psi }/\|\tilde{\psi }\|$. This projection simply normalizes the magnitude of a point $\tilde{\psi }\in \Psi^{\prime }\subset L^{2}[0,1]$ to result in the closest point on the positive orthant of the unit sphere, *ψ* ∈ *Ψ*. Thus, $\Pi \left({\tilde{\psi }}_{0}\right)={\tilde{\psi }}_{0}/\left\|{\tilde{\psi }}_{0}\right\|$ is the phase kriging estimator of *ψ*_0_ based on a linear estimator ${\tilde{\psi }}_{0}\in \Psi^{\prime }$. Such an estimator represents an extrinsic choice that uses a natural embedding of *Ψ* into *L*^2^ [0, 1] via *Ψ*′.

Let $\Delta_{n}^{+}≔\left\{{\left(x_{1},\ldots ,x_{n}\right)}^{T}\in R^{n}|x_{i}>0,\;\sum_{i=1}^{n}x_{i}=1\right\}$. With the estimated phase components ${\hat{\psi }}_{i}$, the phase kriging estimate of *ψ*_0_ in *Ψ*′ is defined as

(12) $${\tilde{\psi }}_{0}(t)=\overset{n}{\underset{i=1}{\sum }}\zeta_{i}{\hat{\psi }}_{i}(t),$$

where $\zeta ={\left(\zeta_{1},\ldots ,\zeta_{n}\right)}^{T}\in \Delta_{n}^{+}$ minimizes the conditional phase prediction error functional

(13) $$\zeta \mapsto \text{E}\left({\left\|{\tilde{\psi }}_{0}-\psi_{0}\right\|}^{2}|𝒮\right).$$

Positivity of *ζ*_*i*_ is required to ensure that the resulting phase prediction ${\tilde{\psi }}_{0}$ is positive, i.e., the corresponding ${\tilde{\gamma }}_{0}$ is strictly increasing. As with the amplitude kriging estimate in Proposition 1, the following result describes how the vector *ζ* can be computed, again under the idealized setting where the {*γ*_*i*_} can be exactly recovered.

**Proposition 2.** *Assume that there exists a template q such that* ${\hat{\gamma }}_{i}=\gamma$. *Then, the vector* $\zeta \in \Delta_{n}^{+}$ *can be obtained by minimizing* *ζ* *↦* *ζ*^*T*^ *𝒱*_*p*_*ζ*, *where the* *n* × *n* *matrix* *𝒱*_*p*_ *contains as its elements* *V*_*p*_(*h*_0*j*,*ω*_) + *V*_*p*_(*h*_*i*0,*ω*_) − *V*_*p*_(*h*_*ij*,*ω*_) *with* $h_{ij,\omega }=\sqrt{{\left\|s_{i}-s_{j}\right\|}_{2}^{2}+\omega \cdot d_{sh}{\left(q_{i},q_{j}\right)}^{2}}(i,j=0,1,\ldots ,n)$. *The phase predictor in* (12) *thus depends only on the conditional phase trace-variogram* *V*_*p*_(*h*_*ω*_).

The proofs of Propositions 1 and 2 are presented in Appendix B of the supplement.

Computation of distances *h*_0*j,ω*_, *j* = 1, …, *n* and *h*_*i*0*,ω*_, *i* = 1, …, *n* relies on the knowledge of the function shape at *s*_0_; for this, we use the shape of the amplitude kriging estimate ${\tilde{q}}_{0}$ at *s*_0_. This aspect of phase kriging reflects the relative nature of phase, as described in Section 3.1, with respect to ${\tilde{q}}_{0}$.

**Remark 3.** An alternative to the proposed extrinsic approach, which we do not consider here, is to construct an intrinsically defined kriging estimator defined directly on *Ψ* using the geometry of the positive orthant of the Hilbert sphere (see, e.g.,Section 7.5.4 of Srivastava and Klassen (2016)). For example, a phase predictor can be defined as a weighted Karcher mean via the intrinsic distance on *Ψ*, ${\tilde{\psi }}_{0}=\text{arg }{\text{min}}_{\psi \in \Psi }\sum_{i=1}^{n}\zeta_{i}{\text{cos}}^{-1}{\left(\langle \psi ,\psi_{i}\rangle \right)}^{2}$, where $\zeta ={\left(\zeta_{1},\ldots ,\zeta_{n}\right)}^{T}\in \Delta_{n}^{+}$ is the minimizer of $\text{E}\left({\text{cos}}^{-1}{\left(\langle {\tilde{\psi }}_{0},\psi_{0}\rangle \right)}^{2}|𝒮\right)$, i.e., the intrinsic counterpart to the extrinsic conditional phase prediction error specified in (13). Unfortunately, Proposition 2 does not hold in this case. In particular, without linearity as in the extrinsic approach, the prediction error cannot be decomposed as a function of the conditional phase trace-variogram. This, in turn, prohibits direct estimation of ${\tilde{\psi }}_{0}$.

### 4.3. Final prediction via combination of amplitude and phase kriging estimates

The predicted amplitude and phase kriging estimates ${\tilde{q}}_{0}$ and ${\tilde{\psi }}_{0}$ include all information about the magnitude, shape and temporal characteristics of the final prediction, but not the translation, which is lost due to the square-root slope transformation. To account for this, we use the starting points *f*_*i*_(0) (*i* = 1, …, *n*) of the observed functions and apply ordinary kriging (Cressie and Wikle, 2011) to obtain a translation prediction ${\hat{T}}_{0}$ of the function *f*_0_.

Recall the inverse of the square-root slope transformation *Q*^−1^ : (*R* × 𝒬) → ℱ from Section 2. The final kriging estimate combines the three estimates of amplitude, phase and translation as follows. First, we combine the amplitude and phase predictions using $q_{0}^{*}=\left({\tilde{q}}_{0},{\tilde{\gamma }}_{0}^{-1}\right)$, where ${\tilde{\gamma }}_{0}(t)=\int_{0}^{t}\Pi {\left({\tilde{\psi }}_{0}\right)}^{2}(u)du$ is the phase prediction. The combined kriging estimate of *f*_0_ at site *s*_0_ then is $f_{0}^{*}=Q^{-1}\left(q_{0}^{*},{\hat{T}}_{0}\right)$, where ${\hat{T}}_{0}$ is the predicted starting point.

## 5. Amplitude-phase clustering

Amplitude and phase distances arising from amplitude-phase separation enable separate distance-based amplitude and phase clustering of functional data. Spatially informed adaptations can now be defined through the use of dissimilarity measures by combining the amplitude (phase) distance and amplitude (phase) trace-variogram. Incorporating amplitude-phase separation into clustering can lead to more interpretable clusters. For example, in the famous Canadian weather data (Ramsay and Silverman, 2005) considered in Section 7.2, we note that daily average temperatures at sites with similar extreme temperatures (similar amplitude) need not experience similar seasonal trends. Thus, one would reasonably expect different clustering results corresponding to the two components.

In contrast to clustering independent data, detecting homogeneous partitions of spatially correlated objects must additionally account for spatial dependence by grouping them based on both their similarity as well as proximity on the spatial domain. The proposed amplitude-phase clustering approach can be viewed as an extension of the spatially informed adaptations of clustering and classification for multivariate data (Oliver and Webster, 1989; Bourgault et al., 1992). While several distance-based clustering approaches can be used, we consider hierarchical clustering based on spatially-weighted dissimilarity matrices (Giraldo et al., 2012), by combining the amplitude (phase) distance and amplitude (phase) trace-variogram, and generate spatially-informed amplitude and phase clusters separately. Given observations {*q*_*i*_, *s*_*i*_ ∈ 𝒟} (*i* = 1, …, *n*), the amplitude and phase dissimilarity matrices are defined as

(14) $$d_{A,ij}=d_{a}\left(q_{i},q_{j}\right)\times V_{a}\left(\left\|s_{i}-s_{j}\right\|\right),\;d_{P,ij}=d_{p}\left(q_{i},q_{j}\right)\times V_{p}\left(h_{ij,\omega }\right),\;i,j=1,\ldots ,n,$$

respectively. Since the dissimilarity matrices measure the discrepancy in amplitude and phase for each pair of functions, it is not necessary to choose a common template for all of the functions for alignment. Instead, we simply choose one of the functions in each pair as a template to compute the amplitude and relative phase distances between them. This ensures that ${\hat{V}}_{a}$ and ${\hat{V}}_{p}$ expressed in a simplified form via pairwise distances in (9) can be used. In the implementation of hierarchical clustering, we use complete linkage to define the discrepancy between clusters. The number of clusters is chosen by minimizing the average silhouette (Rousseeuw, 1987), which quantifies the difference in similarity of an object to its own cluster versus other clusters.

Because the trace-variogram is an increasing function of the distance *h* (or *h*_*ω*_), clustering based on dissimilarities in (14) tends to generate partitions with good spatial contiguity in the presence of strong spatial dependence. Further, it is evident that clustering results do not depend on the overall scale of the trace-variogram, but rather its structure (rate of increase), which reflects the spatial correlatedness among the amplitude (phase) components. In particular, when there is no spatial dependence, the trace-variogram is constant, and the dissimilarity measures in (14) simplify to the amplitude and phase distances (multiplied by a different constant in each case). Furthermore, it is evident that hierarchical clustering based on the amplitude and phase dissimilarity measures defined in (14) are invariant to a global scaling of the amplitude and phase trace-variograms *V*_*a*_ and *V*_*p*_. Appendix D in the supplement contains a more detailed discussion of this property.

## 6. Simulations

In the simulation studies, we assess the performance of the proposed amplitude-phase kriging and clustering methods. The fitting of variograms is carried out using functions in the R packages geofd (Giraldo et al., 2020) and geoR (Ribeiro Jr et al., 2020). Joint kriging and alignment in Algorithm 1 is carried out by appropriately modifying the relevant functions in the R package fdasrvf (Tucker, 2021). Hierarchical clustering is performed using the hclust function in R. All core computing tasks in this paper were conducted using a high performance computing cluster.

### 6.1. Kriging performance

#### 6.1.1. Simulated data

We fix the spatial locations to equally-spaced sites on a 5 × 5 grid with *x*, *y* coordinates taking the values (−2, −1, 0, 1, 2). Spatial functional data *f*_*i*_ (*i* = 1, …, 25) is generated using the model

$$f_{i}\left(\gamma_{i}(t)\right)=\overset{K}{\underset{j=1}{\sum }}a_{i,j}\phi_{j}(t)+e_{i}(t),\;t\in [a,b],$$

on the original function space ℱ (and not the SRSF transformed space 𝒬), where, for each *j*, the coefficient vector [*a*_1,*j*_, …, *a*_25,*j*_] follows a multivariate normal distribution with a specific mean vector *θ* and the Matérn covariance $C_{\text{Mat}}\left(\cdot ,\cdot ;\sigma_{a}^{2},0.5,\ell_{1}\right)$; here, $\sigma_{a}^{2}$ is the scale parameter, *ℓ*_1_ is the range, and the smoothing parameter is fixed to 0.5. This imposes spatial correlation in the amplitude component of the simulated data. Holding *i* fixed, the coefficients for the basis *ϕ*_*j*_, *j* = 1, …, *K* are assumed to be independent. We consider two simulation settings based on the choice of basis functions:

1. *Bimodal*: set *K* = 1 and *ϕ*_1_(*t*) = − cos(2*πt*) on [*a*, *b*] = [−1, 1], with the mean vector *θ* of [*a*_1,1_, …, *a*_25,1_] identically set to 5;
2. *B-spline*: set *K* = 10 and ${\left\{\phi_{j}\right\}}_{j=1}^{K}$ to be cubic B-splines on [*a*, *b*] = [0, 1] with the mean vector *θ* for each *i* equal to (1, 2, 3, 4, 5, 5, 4, 3, 3, 2, 1)^*T*^.

We now describe how spatial correlation is induced amongst the warping functions. The phase components *γ*_*i*_, *i* = 1, …, 25 are chosen to be the cumulative distribution functions of the $\text{Beta}\left(1,e^{b_{i}}\right)$ density with {*b*_1_, …, *b*_25_} generated from the correlated uniform distribution on [−*B*, *B*], by transforming a random sample from the multivariate normal distribution with covariance *C*_*Mat*_(·, ·; 1, 0.5, *ℓ*_2_). The parameter *B* determines the magnitude of phase variation while *ℓ*_2_ controls the range of spatial dependency. Additionally, when the B-spline model is used, we consider two scenarios depending on whether the correlation between phase components depends on the shape of observed spatial functional data:

1. *B-spline Scenario 1*, where spatial phase correlation does not depend on function shapes: we use (6) with *ω* = 0 to induce correlations between warping functions and set *ℓ*_1_ = *ℓ*_2_ = 8^1*/*2^ with *e*_*i*_ generated from a white noise process with variance 0.25;
2. *B-spline Scenario 2*, where spatial phase correlation depends on function shapes: we use (6) with *ω* = 10 to induce correlations between parameters of the warping functions, and set *ℓ*_1_ = 8^1*/*2^ and *ℓ*_2_ as the median of pairwise distances computed via (6).

#### 6.1.2. Comparison with other methods

We compare predictive performance of the proposed amplitude-phase kriging method (APK) to three competing approaches: (1) ordinary kriging without alignment (OK) (Giraldo et al., 2011), (2) universal kriging without alignment (UK) (Menafoglio et al., 2013), and (3) two-stage kriging (TSK). For (3), we align the observed functions using the joint template-based alignment procedure described in Section 2.2, followed by ordinary kriging (Giraldo et al., 2011) applied to the aligned functions in SRSF space. Additionally, a translation prediction is generated in the same way as in amplitude-phase kriging. Then, the SRSF and translation predictions are combined via *Q*^−1^ to yield a prediction in the original function space.

**Performance metrics.** To assess performance, we apply leave-one-out cross-validation. Let *f*^[−*i*]*^ denote the prediction of *f*_*i*_ using all observations except the *i*th, and *q*^[−*i*]*^ and *q*_*i*_ denote their SRSFs; $f_{*}^{\left[-i\right]}$ is $f^{[-i]*}$ after optimal alignment to *f*_*i*_ and $\dot{f}$ is the time derivative of *f*. To measure the accuracy of predictions, we compute the following five error metrics:

- Amplitude least squares: $E1=n^{-1}\sum_{i-1}^{n}{\left\|f_{*}^{\left[-i\right]}-f_{i}\right\|}^{2}$;
- Amplitude Sobolev least squares: $E2=n^{-1}\sum_{i=1}^{n}{\left\|{\dot{f}}_{*}^{\left[-i\right]}-{\dot{f}}_{i}\right\|}^{2}$;
- Amplitude mean squared error: $E3=n^{-1}\sum_{i=1}^{n}d_{a}{\left(q^{[-i]*},q_{i}\right)}^{2}$;
- Phase mean squared error: $E4=n^{-1}\sum_{i=1}^{n}d_{p}{\left(q^{[-i]*},q_{i}\right)}^{2}$;
- *L*^2^ prediction error: $E5=n^{-1}\sum_{i=1}^{n}{\left\|f^{[-i]*}-f_{i}\right\|}^{2}$.

The first three are amplitude errors while the fourth one is the phase error. The last metric is simply based on the standard mean squared error, i.e., the *L*^2^ distance. We use a variety of amplitude/phase error metrics for fair comparison. Note that the mean squared error *E*5 accounts for a combination of amplitude and phase errors and tends to be more sensitive to phase.

**Results.** The advantages of amplitude-phase kriging over other methods are summarized in Table 1; the table reports average prediction errors (with standard deviations in parentheses) over 50 simulation runs. Best performance is highlighted in bold. Compared to ordinary and universal kriging, the improvement in amplitude errors is large when significant phase variation is present in the data. Although the two-stage method has similar performance to the proposed method in terms of amplitude prediction, amplitude-phase kriging shows a clear advantage in predicting the phase of target functions (*E*4).

Data simulated using the B-spline basis (under both scenarios) exhibits higher shape variation than the bimodal case, and represents the more challenging setting for both amplitude-phase kriging and the two-stage method. Nonetheless, amplitude-phase kriging outperforms ordinary and universal kriging in most cases, even when phase variation is small. In particular, when the phase components are dependent on function shapes, amplitude-phase kriging has a clear advantage in terms of the amplitude errors *E*2 and *E*3, and the phase error *E*4. Further, amplitude-phase kriging yields smaller amplitude and phase errors than two-stage kriging in this case; this is due to the spatially-informed alignment via Algorithm 1.

While the proposed approach does not outperform ordinary or universal kriging in terms of the *L*^2^ prediction error *E*5, it has been noted in Srivastava and Klassen (2016) that the *L*^2^ distance, which is used to define this error metric, is not a good measure of amplitude and/or phase differences. Furthermore, since ordinary and universal kriging are optimal under the *L*^2^ metric, the results based on these measures are naturally biased towards these methods. The amplitude-phase kriging errors are mainly due to phase prediction, which is especially challenging on the boundary of the spatial domain since fewer neighbors are available.

**Remark 4.** In the simulated setting involving bimodal data, the performance of two-stage kriging is very similar to that of the proposed amplitude-phase kriging method, especially in terms of the amplitude errors *E*1, *E*2, and *E*3. The data in this case follows a one-dimensional model, and as such, the shapes of all functions are the same across the entire spatial domain. Thus, the global Karcher mean is very similar to the proposed spatially-weighted template.

**Fig. 4.**
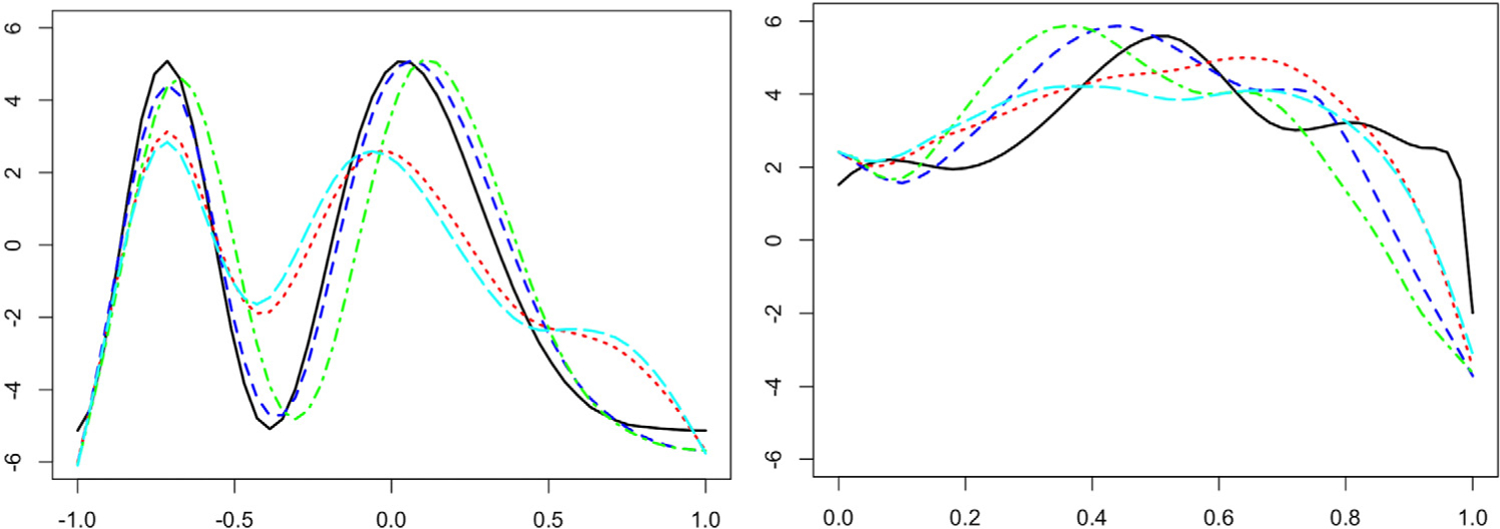
Example predictions obtained via amplitude-phase kriging (blue), ordinary kriging (red), two-stage kriging (green) and universal kriging (cyan). Left: Bimodal simulation with *B* = 1. Right: B-spline Scenario 1 simulation with *B* = 1. The true function is in black.

Fig. 4 shows predictions generated by the four different methods for a single target function based on the bimodal simulation with *B* = 1 (left), and the B-spline Scenario 1 simulation with *B* = 1 (right). In general, ordinary and universal kriging fail to capture important features of functions in the predictions when phase variation is present in the data. In the left panel, the ordinary and universal kriging predictions severely underestimate the two peaks and the valley. In the right panel, the two methods yield predictions that are ‘flat’ over a large portion of the domain and fail to capture any of the shape patterns in the true function. The two-stage method appears to perform relatively well in terms of amplitude prediction, but does not provide a viable phase prediction. The proposed amplitude-phase kriging, on the other hand, successfully captures prominent function features, as well as their magnitude, and provides satisfactory phase predictions. These improvements often result in significant decreases in the various amplitude and phase error metrics. Appendix A in the supplement reports results of another simulation study that directly explores the benefits of spatially-informed alignment in amplitude-phase kriging.

**Fig. 5.**
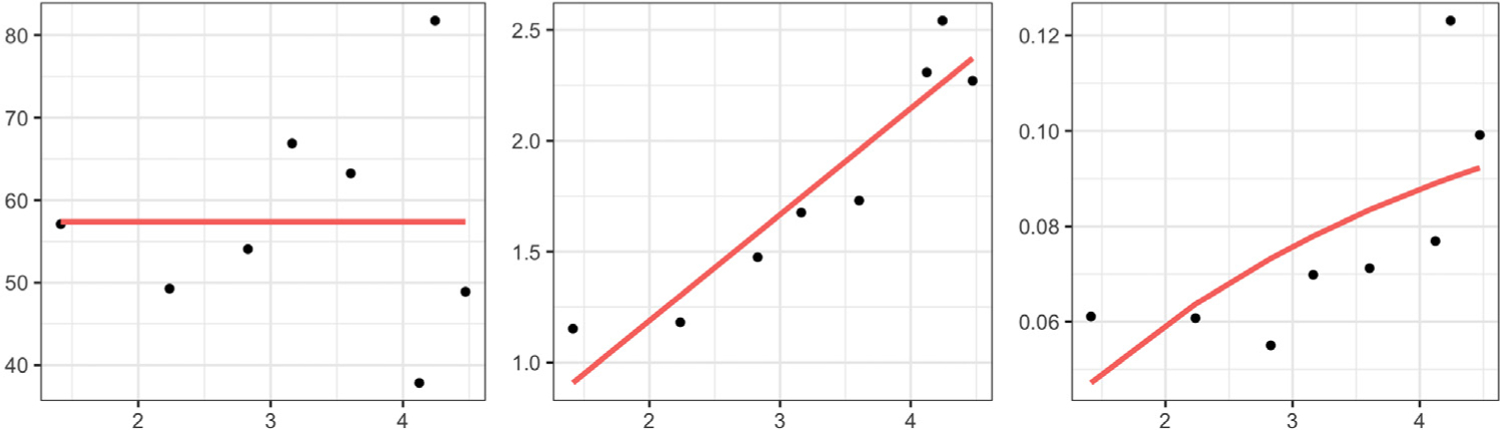
Estimation of trace-variograms for prediction at site 13 under the Bimodal simulation scenario with *B* = 1; site 13 was left out and the rest of the observations were used to estimate the trace-variograms. The dots represent the empirical *L*^2^ (ordinary kriging), amplitude and phase trace-variograms. Estimates of the trace-variograms (red curves) were obtained by fitting a Matérn variogram model to the empirical trace-variograms. For the phase trace-variogram, the estimated optimal value of the tuning parameter *ω* is 0.

**Fig. 6.**
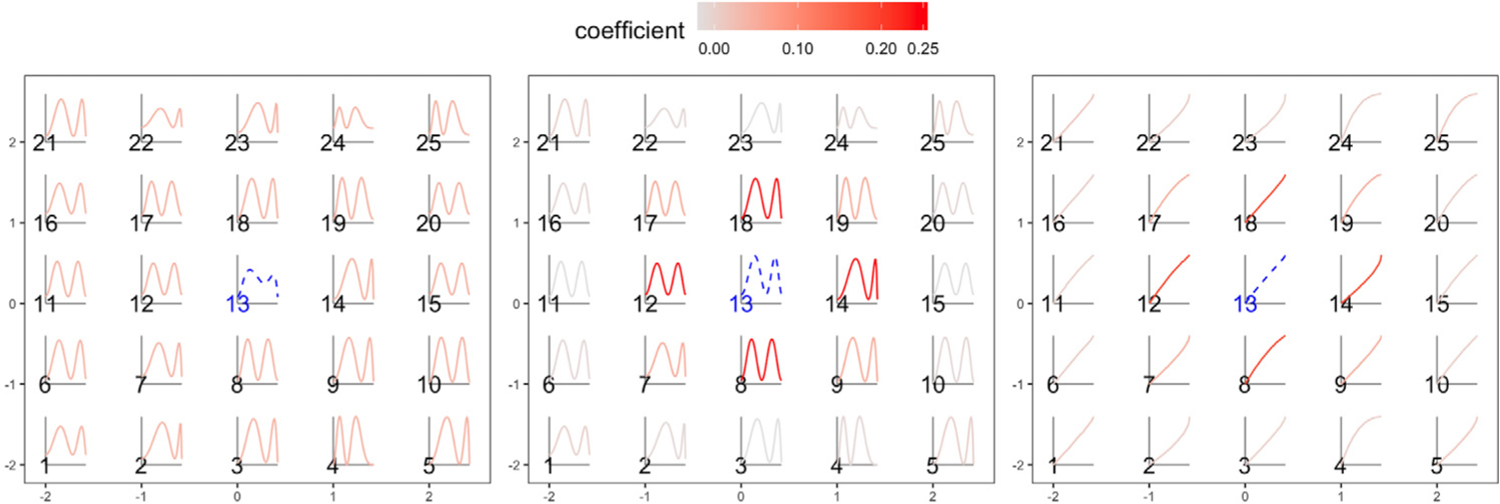
Ordinary (left), amplitude (middle) and phase (right) kriging maps for prediction at site 13 under the bimodal simulation scenario with *B* = 1. The magnitude of kriging coefficients to construct the estimators (dashed blue) are shown in red.

We further illustrate why amplitude-phase kriging yields better predictions than ordinary kriging in the presence of phase variation. Here, we use a single simulation run for the bimodal scenario with *B* = 1. Fig. 5 displays the empirical *L*^2^ (left), amplitude (middle) and phase (right) trace-variograms; the fitted Matérn models are shown in red. In Fig. 6, we show the magnitude of optimal kriging coefficients for the observed data when trying to predict at site 13. Again, we consider ordinary, amplitude and phase kriging in the left, middle and right panels, respectively. Due to the ‘flat’ estimate of the *L*^2^ trace-variogram, ordinary kriging assigns very similar coefficients to all of the observed functions, i.e., it fails to capture the spatial dependence in the data. On the other hand, amplitude and phase kriging result in reasonable coefficient estimates: observations in the spatial neighborhood of site 13 have largest kriging coefficients due to the strong spatial dependence in the data. The resulting estimators are shown in dashed blue at site 13. It is evident that the ordinary kriging prediction underestimates the magnitude of the two extrema; the amplitude kriging prediction is much better at capturing these features. This result is similar to the one presented in the left panel of Fig. 4. Since all of the functions in the bimodal simulation scenario have the same shape, the estimated optimal value of the tuning parameter *ω* is 0. Thus, function shapes do not contribute to the phase trace-variogram and phase prediction. We provide a similar set of results for the B-spline scenario with irregularly-spaced sites in Appendix E in the supplement; the findings are very similar.

### 6.2. Clustering

#### 6.2.1. Simulated data

Let *n* denote the number of spatial sites where data was observed and *I* the number of clusters. Then, $n=\sum_{i=1}^{I}n_{i}$, where *n*_*i*_ is the number of functions in cluster *i*. Motivated by the fact that amplitude and phase in real data scenarios may exhibit different clustering patterns, we simulate the true partitions with respect to amplitude and phase separately. Our aim is to validate that the proposed amplitude-phase clustering method is able to reveal the true underlying partitions of both amplitude and phase simultaneously, irrespective of whether the spatial partitions of each component agree.

**Fig. 7.**
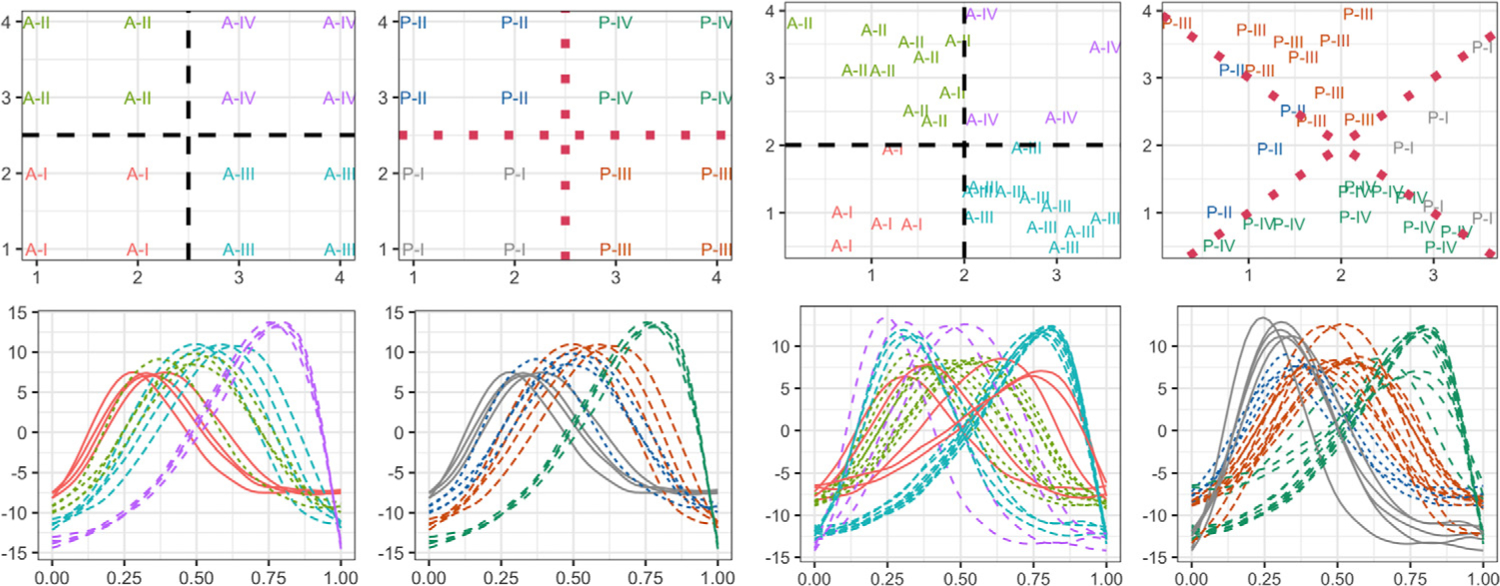
Top row: Ground truth amplitude (column 1) and phase (column 2) partitions for the agree design with spatial sites on a 4 × 4 grid with integer coordinates. Ground truth amplitude (column 3) and phase (column 4) partitions for the disagree design with uniformly sampled spatial sites on the domain [0, 4]^2^. Black dashed lines delineate the boundaries of the amplitude clusters, while red dotted lines delineate the boundaries of the phase clusters. Bottom row: Example dataset generated for the agree and disagree designs, with colors corresponding to the true amplitude or phase clusters displayed in the top row.

We consider two different designs: (i) where amplitude and phase cluster partitions are the same (agree), and (ii) where they are not (disagree). For (i), sites are on a 4 × 4 grid with integer coordinates 1, 2, 3, 4, and are partitioned into four equally sized clusters via the lines *x* = 2.5 and *y* = 2.5. For (ii), 30 sites are chosen uniformly on [0, 4]^2^; the amplitudes are partitioned by the lines *x* = 2 and *y* = 2, while the phases are partitioned by the lines *y* = *x* and *y* = 4 − *x*. The top row in Fig. 7 displays the two designs: the left two panels correspond to the ground truth amplitude and phase partitions for the agree design, respectively, while the right two panels display the same for the disagree design. In the bottom row, we display one example of simulated data for these two designs. The colors in each panel correspond to the ground truth clustering according to amplitude or phase.

Let *f*_*ij*_ be the *j*th functional observation in cluster *i*. We generate spatial functional data with domain [0, 1] as *f*_*ij*_(*t*) = {(*a*_*ij*_*μ* + *e*_*ij*_) ◦ *γ*_*ij*_} (*t*) (*i* = 1, …, *I*; *j* = 1, …, *n*_*i*_). We set *μ*(*t*) = − cos(2*πt*), *a*_*ij*_ = *iδ*_*a*_ + *ϵ*_*a,ij*_, and *γ*_*i*_ as the cumulative distribution function of $\text{Beta}\left(1,e^{b_{ij}}\right)$, where *b*_*ij*_ = *iδ*_*b*_ + *ϵ*_*b,ij*_; *δ*_*a*_ and *δ*_*b*_ are fixed parameters that control the amplitude and phase differences between clusters, respectively. The vector {*ϵ*_*a,ij*_} is generated from a multivariate normal distribution with a mean vector (5, …, 5)^*T*^ and Matérn covariance $C_{\text{Mat}}\left(\cdot ,\cdot ;\sigma_{a}^{2},0.5,\ell \right)$. The vector {*ϵ*_*b,ij*_} follows the correlated uniform distribution on [−*B*, *B*]^*n*^ with the same correlation range *ℓ*; *e*_*ij*_ is a zero mean Gaussian process with a diagonal covariance. We fix $\sigma_{a}^{2}=1$, *B* = 1, *σ*_*e*_ = 0.5 and *ℓ* = 8^1*/*2^. In the bottom row of Fig. 7, we display one example of simulated data for the agree and disagree designs. The colors in each panel correspond to the ground truth clustering according to amplitude or phase.

#### 6.2.2. Comparison with another method

We repeat each simulation 100 times, and compare amplitude-phase clustering (APC) to the *L*^2^ distance-based method (*L*^2^*C*) (Giraldo et al., 2012) using the rand index (Rand, 1971).

**Fig. 8.**
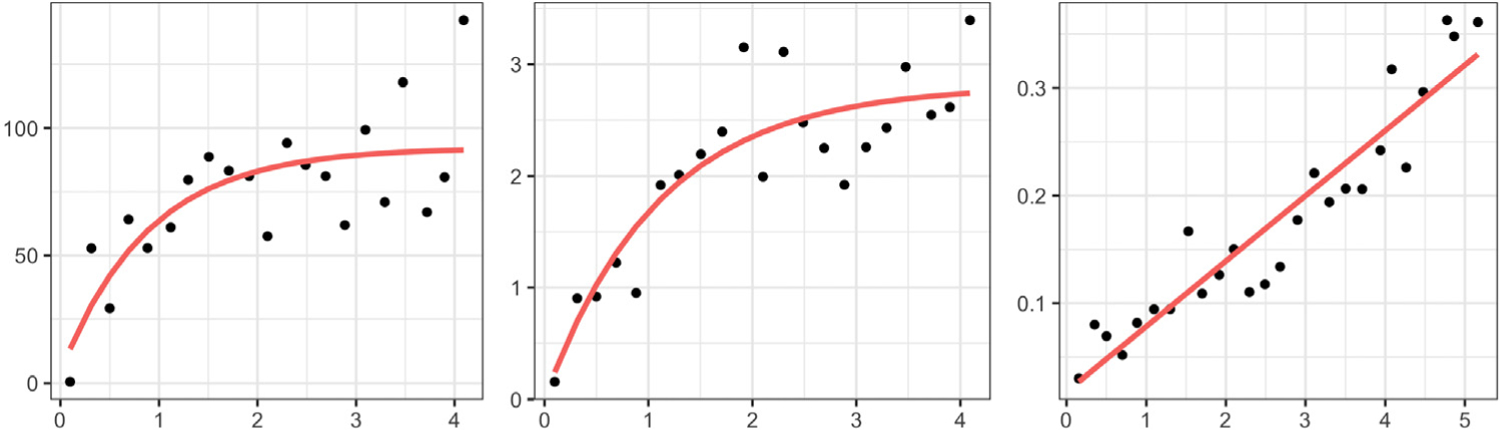
Estimation of trace-variograms for clustering under the disagree design with *δ*_*a*_ = 2 and *δ*_*b*_ = 0.5. The dots represent the empirical *L*^2^, amplitude and phase trace-variograms. Estimates of the trace-variograms (red curves) were obtained by fitting a Matérn variogram model to the empirical trace-variograms.

**Results.** The means and standard deviations of the rand indices for each design, and different choices of *δ*_*a*_ and *δ*_*b*_, are shown in Table 2; best performance is highlighted in bold. The proposed amplitude-phase clustering approach outperforms the *L*^2^ distance-based method in most scenarios, even when the amplitude and phase partitions agree. When the true partitions are different, the amplitude-phase clustering is far superior, especially for the larger values of *δ*_*a*_ and *δ*_*b*_. The *L*^2^-based approach is always forced to compromise between the true amplitude and phase clusters, while the proposed approach treats them separately. Further, the *L*^2^ metric is sensitive to phase differences. As a result, when *δ*_*b*_ is large, it captures the phase clustering and exhibits similar performance to the proposed method in that regard. However, it is unable to recover the true amplitude clusters. Fig. 8 shows the empirical and fitted trace-variograms for particular values of *δ*_*a*_ and *δ*_*b*_, and demonstrates that spatial dependence is captured by all variograms, increasing the chance of grouping the subjects with stronger spatial correlation.

## 7. Real data analysis

### 7.1. Kriging of daily ozone data in north california

We apply the proposed amplitude-phase kriging method to U.S. daily ozone data, available on the air data website^1^ of the United States Environmental Protection Agency. We focus on a small area in North California (35° ~ 39° N, 120 ~ 123° W) with 24 observation stations. Each station recorded daily average ozone concentration (parts per million) for the year 2018. We smooth the data using splines with smoothing parameter *ι* = 3 × 10^−4^. We evaluate the effect of smoothing on kriging performance in Appendix C in the supplement.

#### 7.1.1. Results

We use leave-one-out cross-validation on the 24 smoothed observations and report the mean of the five error metrics, *E*1–*E*5, for ordinary kriging (OK), two-stage kriging (TSK) and amplitude-phase kriging (APK) in Table 3; best performance is highlighted in bold. We do not compare to universal kriging here since this approach focuses on kriging residual functions after accounting for a spatially varying mean function. The proposed method outperforms ordinary kriging in terms of the reported amplitude/phase error metrics *E*2, *E*3 and *E*4. The amplitude and phase mean squared errors (*E*3 and *E*4) of amplitude-phase kriging are 13% and 8.6% smaller, respectively, compared to ordinary kriging. This shows that combining separate amplitude and phase predictions has a clear advantage in real data scenarios. Compared to two-stage kriging, amplitude-phase kriging generates more accurate amplitude predictions as evidenced by smaller *E*2 and *E*3 errors. This is most likely due to moderate shape variation among the spatial functional data. Two-stage kriging outperforms amplitude-phase kriging in terms of the phase error *E*4.

**Fig. 9.**
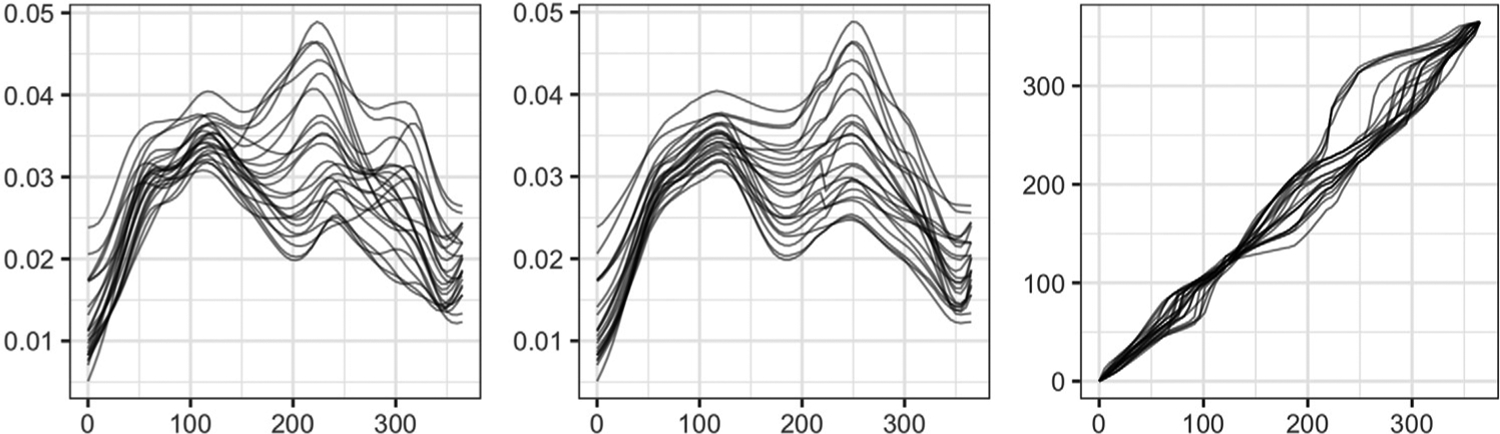
After leaving out the observation at site 8, the remaining ozone concentration functions observed at 23 other sites (left) are aligned to the estimated amplitude prediction at site 8 using Algorithm 1, resulting in separate amplitude (middle) and phase (right) components.

**Fig. 10.**
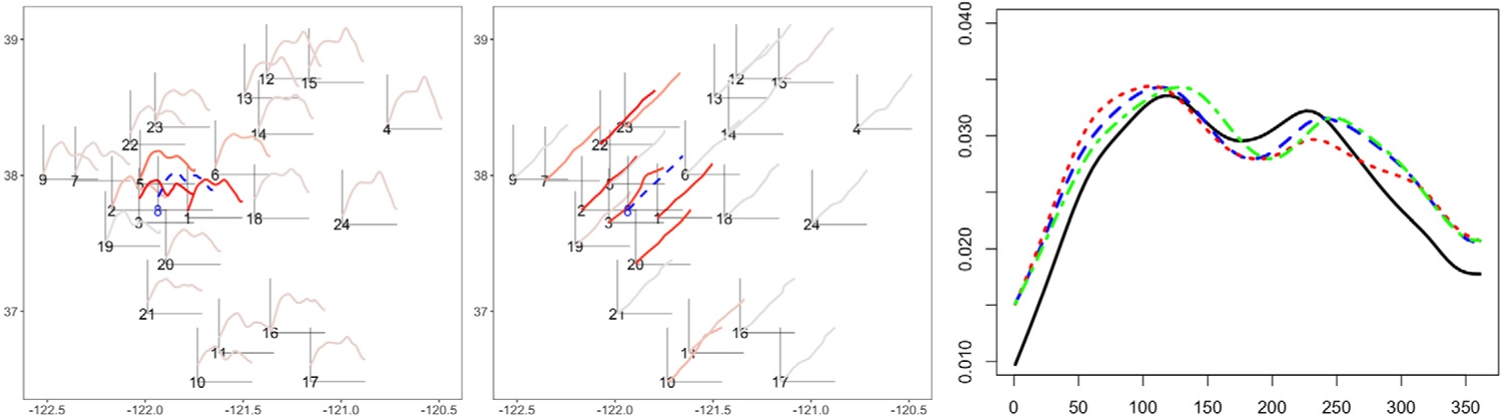
Amplitude-phase kriging of amplitude (left) and phase (middle) components at site 8. The solid curves are estimated amplitude and phase components at observed sites; dashed blue lines are the predicted amplitude and phase components. The color shading shows the contribution (from 0 to 1) from each site to the prediction. Right: The prediction for site 8 with the true function (black) and predictions obtained via amplitude phase-kriging (blue), ordinary kriging (red) and two-stage kriging (green).

Focusing on site 8, we present more detailed alignment and kriging results based on the proposed approach. We present the results of amplitude-phase separation, computed via Algorithm 1, in Fig. 9. The given spatial functional data (except for the datum observed at site 8) is given in the left panel. It is clear that phase variation is present in the sample. The estimated warping functions, with respect to the amplitude kriging predictor at site 8, are shown in the right panel; phase variation in the ozone concentration functions is mainly due to local delays/advances in the timeline, which represent significant deviations from identity warping. The middle panel displays the aligned data. The right panel in Fig. 10 highlights the advantage of amplitude-phase kriging as compared to ordinary kriging: between days 200 and 300, where significant phase variation is present, amplitude-phase kriging is much more effective at predicting the shape of the function at site 8. In particular, ordinary kriging underestimates the magnitude of the second peak of ozone concentration. Accurate prediction of the phase component is difficult in practice since its definition depends on the shape of functional data. From the left and middle panels in Fig. 10, we can see that amplitude kriging generally borrows information from neighboring sites since we only use the spatial coordinates (distance) to model the dependency in this case. On the other hand, in phase kriging, we include both the spatial locations and the shape of the observed functions to model the dependency. Thus, the highest contribution into the final kriging estimate is a combination of phase functions that are spatially nearby, and those that correspond to observed functions that have a similar shape to the predicted amplitude. Furthermore, the spatial dependency in the phase component is generally fairly weak. This is why many previous studies prefer to treat phase variability as noise. However, in this real data analysis, we have found that even if the phase signal is not as strong as the amplitude signal, separate amplitude and phase prediction is still beneficial as evidenced in Table 3 and the right panel in Fig. 10.

### 7.2. Clustering of canadian weather data

We apply the proposed amplitude-phase clustering method to the Canadian weather data (Ramsay and Silverman, 2005). The data can be found in the R package ‘fda’ (Ramsay et al., 2020). In this paper, we analyze the daily temperature data averaged over 1960–1994, collected at 35 stations in Canada. Because the 35 stations cover a large area, we first filter out the longitudinal and latitudinal trends by fitting a functional linear regression model where longitude and latitude are included as covariates; the same approach was taken in Giraldo et al. (2012). The resulting functional residuals are then smoothed using splines (with low smoothing parameter *ι* = 5 × 10^−5^) and used as the data for clustering.

#### 7.2.1. Results

**Fig. 11.**
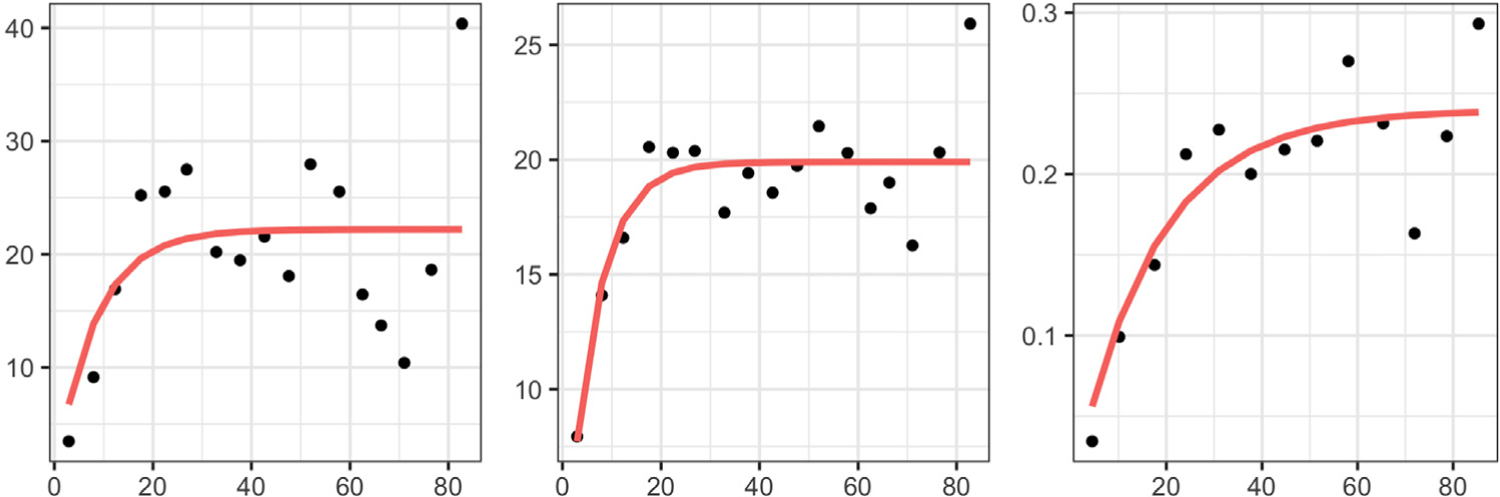
Estimation of trace-variograms for clustering of the Canadian weather data. The dots represent the empirical *L*^2^, amplitude and phase trace-variograms. Estimates of the trace-variograms (red curves) were obtained by fitting a Matérn variogram model to the empirical trace-variograms.

We use the clustering method described in Section 5 and compare the results to the *L*^2^ metric-based clustering of Giraldo et al. (2012). The empirical and fitted *L*^2^, amplitude and phase trace-variograms are shown in Fig. 11. There is evidence of spatial correlations in each of them and the amplitude trace-variogram has a smaller range than the *L*^2^ one. We further observe that the Matérn model fits the empirical amplitude and phase trace-variograms better than the *L*^2^ one, since some quadratic patterns are present in the latter. Values from these fitted variograms are plugged into the dissimilarity measures as weights for clustering.

**Fig. 12.**
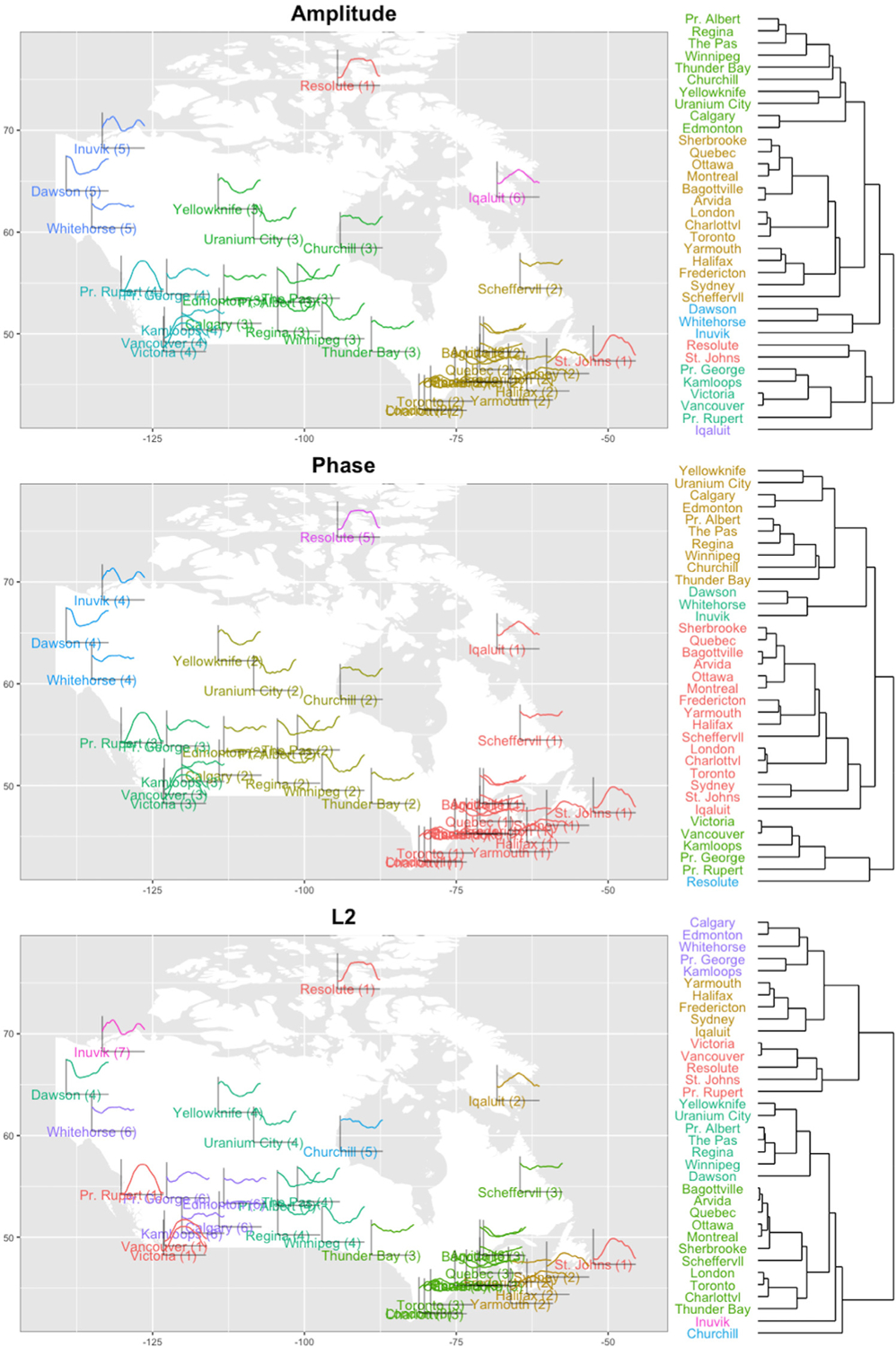
Clustering (average linkage, clusters in different colors) of functional residuals, after adjusting for latitude and longitude effects, obtained from the Canadian weather data.

The hierarchical clustering trees as well as the clustering results on the map of Canada are shown in Fig. 12. Based on separate clustering of amplitude and phase, we discover some interesting results. First, the amplitude and phase clusterings agree in Western and Central Canada. The cities located on the West Coast are further partitioned into South and North clusters, while the cities in the Central region are in a single cluster. Second, the difference between amplitude and phase variation mainly appears in the clustering of Resolute, Iqaluit and St. Johns. Specifically, Resolute and St. Johns are clustered together based on amplitude due to similar magnitude (and shape) of the residual functions whereas Iqaluit is clustered separately. In terms of phase, Iqaluit and St. Johns are included in the large cluster in Southeast Canada, but Resolute forms its own cluster. This is due to a large phase distance between the Resolute residual function and the Iqaluit/St. Johns residual functions: the Resolute function reaches its peak earlier than the other two and has a much longer plateau. Third, compared to the *L*^2^-based clustering method (bottom panel in Fig. 12), amplitude-phase clustering yields fewer clusters and the clusters tend to be more spatially connected. For example, the three cities in the Northwest are clustered together based on amplitude-phase clustering whereas *L*^2^-based clustering separates them into three different clusters. Further, based on *L*^2^-based clustering, we observe an unnatural result: Resolute, a station in the Arctic Circle, is clustered with the Vancouver and Victoria stations on the West Coast and St. Johns on the East Coast. We also applied hierarchical clustering without spatial weighting to the same dataset (see Appendix E in the supplement). It is clear that involving spatial dependency in the clustering helps preserve connectivity of adjacent sites, making the results more interpretable.

## 8. Discussion

It is difficult to verify the key assumptions of stationarity and isotropy for spatial functional data, especially when one decouples amplitude and phase components, which effectively results in two sets of functional data. Despite this, when deviation from stationarity is not too large, the amplitude and conditional phase trace-variograms provide useful summary statistics of spatial variation. Results from simulations and real data analyses offer corroboration. Although some of the presented empirical amplitude and (conditional) phase trace-variograms could indicate a spatial trend in the mean, it is difficult to assess whether the stationarity assumption has in fact been violated. If a spatial trend in the mean is of concern, one can adapt the proposed framework to a universal amplitude-phase kriging approach. This extension is non-trivial as it requires a regression framework for amplitude and phase. For amplitude, the specified regression model must be invariant to warping. For phase, considerable difficulties are posed by the non-Euclidean nature of its representation space. We will consider this extension in future work.

Based on simulations in Section 6, the phase trace-variogram is more informative when allowing for the plausibility that two nearby functions share similar shapes. Thus, a rigorous study of the conditional phase trace-variogram, when conditioned on the shape random field, will add further insight, but is beyond the scope of this paper. The main challenge will be to reconcile the variogram definition with the property that a phase functional random field will almost never satisfy stationarity since it is only interpreted in a *relative* sense.

Evidently, formulating phase variation as an isometric action by the group of warping functions plays a crucial role in this paper. This is enabled by adopting the square-root slope transform *f* → *q*, which maps *f* ◦ *γ* → (*q*, *γ*) under which ∥(*q*, *γ*)∥ = ∥*q*∥. The property that warping or phase variation does not affect the function’s norm drives our definitions of variograms *V*_*a*_ and *V*_*p*_, their estimators, and is used when constructing, and examining properties of, kriging estimates (see proofs of Propositions 1 and 2 in Appendix B in the supplement as well as Proposition 3 and its proof in Appendix C in the supplement). Hence, although several methods for amplitude-phase separation are available, our developments in this paper complement the ubiquity of the *L*^2^ metric in functional data analysis through the use of the square-root slope transform.

Extensions of developments in this paper to the setting of noisy, sparse spatial functional data constitute ongoing work. Variability due to considerable nonparametric or model-based smoothing will then need to be considered in addition to amplitude, phase and spatial variabilities. Promisingly, results here represent the first foray towards analyzing spatial complex functional data objects such as shapes of curves (Srivastava and Klassen, 2016) and surfaces (Jermyn et al., 2017) by decoupling spatial, shape and nuisance variations.

## Supplementary Material

supplement

## Figures and Tables

**Table 1:** Average prediction errors (SD) using metrics *E*1–*E*5, across 50 different replicates, for amplitude-phase kriging (APK), two-stage kriging (TSK), ordinary kriging (OK) and universal kriging (UK). *E*2 is divided by 100 and *E*4 is multiplied by 10 to adjust the scale. *B* controls the magnitude of phase variation.

*Bimodal*						
*B*	Method	*E*1	*E*2	*E*3	*E*4	*E*5
0.5	APK	1.12 (0.33)	**0.46 (0.12)**	**0.55 (0.19)**	**0.15 (0.03)**	10.4 (5.00)
	TSK	**1.11 (0.34)**	**0.46 (0.12)**	0.56 (0.19)	0.18 (0.04)	14.48 (6.56)
	OK	2.34 (1.00)	0.86 (0.32)	0.98 (0.30)	0.17 (0.04)	**8.99 (4.36)**
	UK	2.50 ( 0.99 )	0.91 (0.32)	1.07 (0.33)	0.18 (0.05)	9.20 (4.36)
1	APK	1.48 (0.63)	1.31 (1.19)	**0.81 (0.24)**	0.55 (0.17)	31.42 (14.64)
	TSK	**1.45 (0.59)**	**1.30 (1.15)**	0.82 (0.23)	**0.54 (0.15)**	32.89 (14.11)
	OK	7.94 (3.88)	4.18 (2.19)	4.24 (1.93)	0.88 (0.37)	19.51 (8.05)
	UK	6.86 (2.64)	3.71 (1.86)	3.72 (1.14)	0.83 (0.24)	**19.43 (7.62)**
*B-spline Scenario 1 (independent)*						
*B*	Method	*E*1	*E*2	*E*3	*E*4	*E*5
0.5	APK	1.49 (0.42)	**2.32 (0.99)**	**2.26 (0.44)**	1.00 (0.25)	2.61 (0.57)
	TSK	1.55 (0.45)	2.35 (0.99)	2.36 (0.47)	1.12 (0.32)	3.06 (0.79)
	OK	**1.20 (0.21)**	2.34 (0.97)	2.48 (0.52)	**0.99 (0.22)**	**1.84 (0.29)**
	UK	1.32 (0.22)	2.44 (0.97)	2.67 (0.57)	1.05 (0.25)	1.99 (0.30)
1	APK	1.63 (0.42)	**2.96 (1.82)**	**2.52 (0.53)**	**1.23 (0.28)**	3.65 (0.95)
	TSK	1.63 (0.42)	3.00 (1.77)	2.61 (0.55)	1.39 (0.34)	4.09 (1.09)
	OK	**1.50 (0.27)**	3.28 (1.74)	3.12 (0.68)	1.40 (0.22)	**2.71 (0.65)**
	UK	1.59 (0.29)	3.32 (1.58)	3.17 (0.67)	1.43 (0.26)	2.83 (0.63)
*B-spline Scenario 2 (dependent)*						
*B*	Method	*E*1	*E*2	*E*3	*E*4	*E*5
0.5	APK	1.53 (0.46)	**2.26 (0.95)**	**2.30 (0.42)**	**1.04 (0.25)**	2.85 (0.64)
	TSK	1.56 (0.46)	2.31 (0.95)	2.37 (0.44)	1.17 (0.34)	3.26 (0.75)
	OK	**1.27 (0.24)**	2.39 (0.99)	2.62 (0.60)	1.06 (0.27)	**2.12 (0.49)**
	UK	1.36 (0.24)	2.48 (1.02)	2.74 (0.61)	1.10 (0.27)	2.23 (0.48)
1	APK	**1.66 (0.47)**	**2.96 (1.64)**	**2.55 (0.49)**	**1.34 (0.27)**	4.27 (1.42)
	TSK	1.70 (0.52)	2.99 (1.55)	2.66 (0.52)	1.44 (0.34)	4.44 (1.42)
	OK	1.77 (0.59)	3.70 (1.82)	3.64 (1.10)	1.66 (0.36)	**3.34 (1.05)**
	UK	1.74 (0.39)	3.52 (1.68)	3.46 (0.78)	1.61 (0.32)	**3.34 (1.02)**

**Table 2:** Average rand indices (SD) for estimated partitions based on amplitude-phase clustering (APC) and *L*^2^-based clustering (*L*^2^C), with respect to the true amplitude and phase clusters.

*δ*_*a*_	*δ*_*b*_	Method	Agree		Disagree	
			Amplitude	Phase	Amplitude	Phase
1	0.1	APC	**0.828 (0.106)**	0.751 (0.101)	**0.808 (0.107)**	**0.711 (0.088)**
		*L*^2^C	0.772 (0.079)	**0.772 (0.079)**	0.731 (0.085)	**0.711 (0.067)**
	0.5	APC	0.870 (0.091)	**0.958 (0.052)**	**0.752 (0.086)**	**0.887 (0.083)**
		*L*^2^C	**0.910 (0.072)**	0.910 (0.072)	0.701 (0.046)	0.877 (0.07)
2	0.1	APC	**0.945 (0.067)**	0.767 (0.104)	**0.916 (0.076)**	0.708 (0.087)
		*L*^2^C	0.808 (0.082)	**0.808 (0.082)**	0.779 (0.085)	**0.742 (0.068)**
	0.5	APC	**0.949 (0.071)**	**0.955 (0.059)**	**0.835 (0.085)**	**0.879 (0.087)**
		*L*^2^C	0.908 (0.080)	0.908 (0.080)	0.707 (0.055)	0.873 (0.071)

**Table 3:** Leave-one-out cross-validation average prediction errors of amplitude-phase kriging (APK), two-stage kriging (TSK) and ordinary kriging (OK) for the ozone data in North California. All values were multiplied by 1000.

Method	*E*1	*E*2	*E*3	*E*4	*E*5
APK	4.88	**7.57**	**1.94**	49.02	7.17
TSK	4.67	8.79	2.21	**45.9**	6.6
OK	**4.17**	8.01	2.23	53.64	**6.44**

## Footnotes

1 https://www.epa.gov/outdoor-air-quality-data.
