# Supplementary material for: Variograms for kriging and clustering of spatial functional data with phase variation
Source: Spat Stat. Author manuscript; Available in PMC 2023 Feb 10. (PMC9912960; doi:10.1016/j.spasta.2022.100687)
Supplement: supplement [file NIHMS1867417-supplement-supplement.pdf]

Supplemental Material for ‘Variograms for Kriging and Clustering of Spatial Functional Data with Phase Variation’

Xiaohan Guo[a], Sebastian Kurtek[a] and Karthik Bharath[b]

[a]Department of Statistics, The Ohio State University, 1958 Neil Avenue, Columbus, OH 43210, USA
[b]School of Mathematical Sciences, University of Nottingham, University Park, Nottingham NG7 2RD, UK

ABSTRACT

This file contains supplemental material to support the work presented in ‘Variograms for Kriging and Clustering of Spatial Functional Data with Phase Variation’ by Xiaohan Guo, Sebastian Kurtek, and Karthik Bharath.

Appendix A Performance of Algorithm 1

We begin with a thorough assessment of the performance of Algorithm 1, which is used to compute the spatially-weighted template that enables simultaneous alignment and estimation of the amplitude and conditional phase trace-variograms in the context of kriging.

Sensitivity to initialization. We simulate spatial functional data at 25 randomly (uniformly) sampled locations on the domain $[-2, 2]^2$ based on the B-spline model with independent shape and phase components (see Section 7.1 in the main article); we let $B = 0.5$ and $\ell_1 = \ell_2 = 8^{1/2}$. The unobserved site is always chosen as $s_0 = (0, 0)$ and a corresponding true function is simulated at this site from the same model. In this simulation, we perform amplitude kriging at s_0 via Algorithm 1 using two different initialization approaches, by (i) choosing the observed function that is spatially closest to s_0 as the initialization of the template, and (ii) randomly choosing the initialization of the template from one of the observed functions irrespective of the spatial proximity to s_0. To account for the random initialization in approach (ii), for each simulation, we randomly choose an initial template nine times, record the spatial distance from s_0 to each of the ten initialization sites (one site for (i) and nine sites for (ii)) and resulting amplitude prediction errors $d_a(q^*, q_0)$ between the predictions q^* and the true function q_0.

Figure 1 summarizes the results over 200 simulation runs via boxplots. On the x-axis, we display binned spatial distances from s_0 to the initialization sites; the amplitude prediction errors are given on the y-axis. The figure suggests that the amplitude predictions are not overly sensitive to initialization of Algorithm 1. It is evident, however, that choosing a nearby (closest) site to s_0 tends to result in smallest amplitude prediction errors, as expected. This justifies the proposed initialization approach specified in Algorithm 1 in the main article.

Global vs. spatially-weighted template estimation. Here, we assess the advantages of performing (spatially) local template estimation via Algorithm 1 as compared to global template estimation via the Karcher mean (see discussion in Section 2.2 in the main article) in the context of amplitude kriging. Recall that amplitude prediction using global alignment refers to first aligning all functions to the estimated amplitude Karcher mean and then constructing the prediction using the (SRSFs of) aligned functions. On the other hand, amplitude prediction using local alignment using Algorithm 1, where an estimated spatially-weighted template is used as the prediction.

Using the B-spline model with independent shape and phase components (Section 7.1 in the main article), we simulate spatial functional data at equally-spaced locations on a 7×7 grid with x, y coordinates taking the values $(-3, -2, -1, 0, 1, 2, 3)$. We then perform (leave-one-out) amplitude kriging for the function at site $(-1, -2)$ based on global (Karcher mean-based) and local (Algorithm 1-based) alignment.

Figure 2 shows a comparison of the estimated Karcher mean (red) and the estimated spatially-weighted template (blue); the true function is shown in blue. For plotting, we used the average of the starting points on all functions as the starting point for the Karcher mean, and the translation kriging estimate as the starting point for the spatially-weighted template. It is clear that the spatially-weighted template is much more similar in amplitude (shape) to the true function than the global Karcher mean.

guo.1280@osu.edu (X. Guo); kurtek.1@stat.osu.edu (S. Kurtek); Karthik.Bharath@nottingham.ac.uk (K. Bharath)
ORCID(s): 0000-0002-0423-9882 (X. Guo)

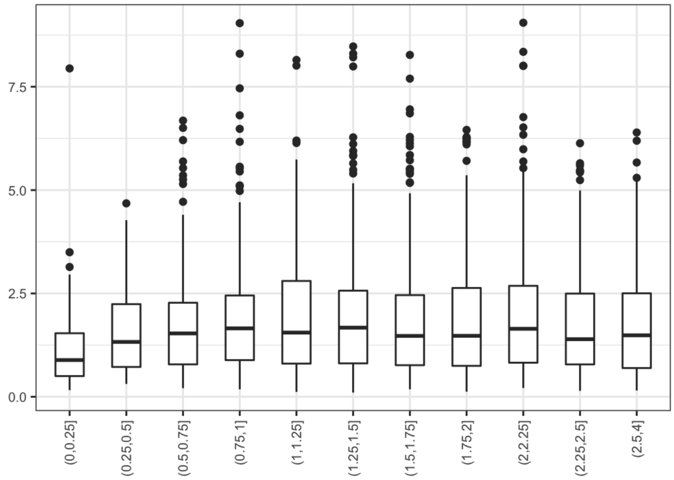

Figure 1: Boxplots of amplitude prediction errors (y-axis) over 200 simulation runs. The binned spatial distances between the unobserved site s_0 and the initialization site are given on the x-axis.

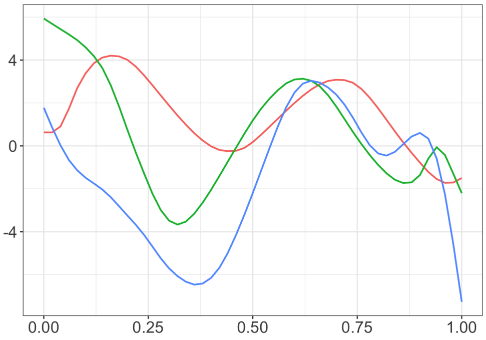

Figure 2: Estimated Karcher mean (red) and spatially-weighted template (green). The true function at site $(-1, -2)$ is shown in blue.

Figure 3 further illustrates pairwise alignment of functions at neighboring sites to the two templates as well as amplitude prediction at site $(-1, -2)$. At location $(-1, -2)$, we show the true function in solid blue, the amplitude prediction constructed via global alignment to the estimated Karcher mean in dashed red, and the spatially-weighted template/prediction in dashed green (same as the green function in Figure 2). We use the true starting point to plot the two predictions in order to highlight amplitude differences between them and the true function. The local alignment-based amplitude prediction shares many of the same shape features as the true blue function; this is not the case for the global alignment-based prediction. In the other eight panels (spatial locations), we show the observed function in blue as well as the alignment of this function to the Karcher mean (red) and to the spatially-weighted template (green). In most cases, the alignment to the spatially-weighted template is much more natural, resulting in an improved amplitude prediction at site $(-1, -2)$.

Convergence. In Figure 4, we empirically assess convergence properties of Algorithm 1 by plotting the prediction errors (top row) and relative change between estimates (bottom row), as a function of iteration number, for three different examples. Each example corresponds to one of the three simulation settings described in the main article: bimodal (left), B-spline with independent amplitude and phase (middle), and B-spline with dependent amplitude and phase (right). To generate the plots, we first calculate the amplitude prediction at site $(0, 0)$ using the data observed on a 5×5 grid with coordinates $(-2, -1, 0, 1, 2)$ (except for $(0, 0)$). The amplitude prediction errors for q_0 at $(0, 0)$, displayed in the first row in Figure 4, are measured via

$$d_a(\hat{q}_0^{(k)}, q_0)^2, \tag{1}$$

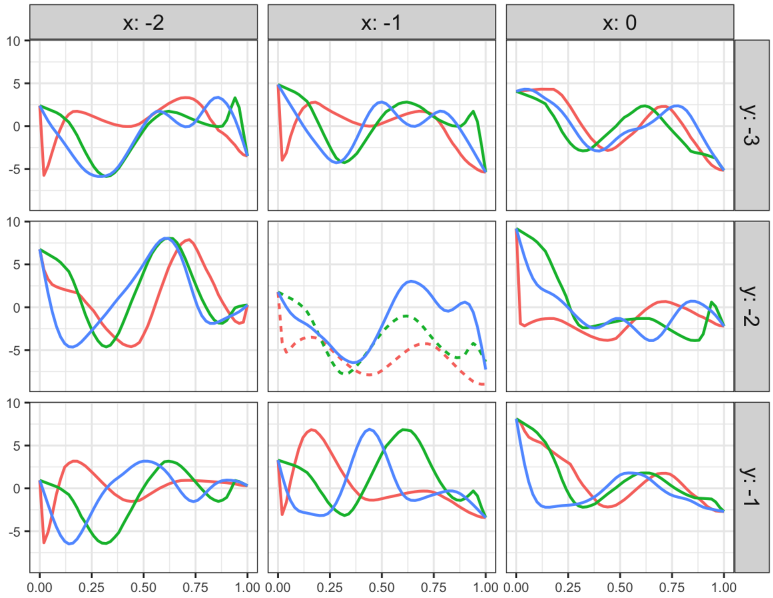

Figure 3: Amplitude kriging at site $(-1,-2)$ and alignment of observed functions at neighboring sites. Each panel represents a single site with labeled coordinates. At site $(-1,-2)$, we show the true function (blue), the Karcher mean-based amplitude prediction (dashed red), and the spatially-weighted template/prediction (dashed green); the starting point for both predictions was fixed to the true starting point. At the other eight sites, we show the observed function (blue), alignment of this function to the Karcher mean (red), and alignment of this function to the spatially-weighted template (green).

where $\hat{q}_0^{(k)}$ is the amplitude prediction at the kth iteration. We also monitor the relative change defined as

$$\|\hat{q}_0^{(k)} - \hat{q}_0^{(k-1)}\| / \|\hat{q}_0^{(k-1)}\|. \tag{2}$$

As seen in the figure, there do not appear to be any convergence issues and the algorithm usually converges in a small number of steps.

Appendix B Proofs of Propositions 1 and 2

Proofs of Propositions 1 and 2. We first prove Proposition 1. Let $\langle \cdot, \cdot \rangle$ denote the L^2 inner-product and $\| \cdot \|$ the corresponding L^2 norm. Then, the prediction error decomposes as follows:

$$E(\|\tilde{q}_0 - (q_0,\gamma_0)\|^2) = \mathrm{E}\Big(\Big\| \sum_{i=1}^{n} \eta_i \{(q_i,\hat{\gamma}_i) - (q_0,\gamma_0)\}\Big\|^2\Big)$$

$$= \mathrm{E}\Big(\sum_{i=1}^{n}\sum_{j=1}^{n} \eta_i\eta_j \langle (q_i,\hat{\gamma}_i) - (q_0,\gamma_0), (q_j,\hat{\gamma}_j) - (q_0,\gamma_0)\rangle \Big)$$

$$= \sum_{i=1}^{n}\sum_{j=1}^{n} \Big(\eta_i\eta_j \int_0^1 \mathrm{E}\big[\{(q_i,\hat{\gamma}_i)(u) - (q_0,\gamma_0)(u)\}\{(q_j,\hat{\gamma}_j)(u) - (q_0,\gamma_0)(u)\}\big] \mathrm{d}u \Big), \tag{3}$$

where the first equality holds due to the constraint $\sum_{i=1}^{n} \eta_i = 1$, and the third equality uses Fubini's theorem. As per our assumption, given a template q, the aligned function $(q_i, \hat{\gamma}_i)$ recovers the underlying amplitude component (q_i, γ) exactly, and we can replace $(q_i, \hat{\gamma}_i)$ by (q_i, γ_i). Under the second order stationary and isotropic assumptions on the amplitude random field $\{(q_s, \gamma_s)\}$, $E[(q_i, \gamma_i)] = \mu_q$ for any i. Thus, the integral in (3) equals $\int_0^1 \mathrm{E}[\{(q_i, \gamma_i)(u) -$

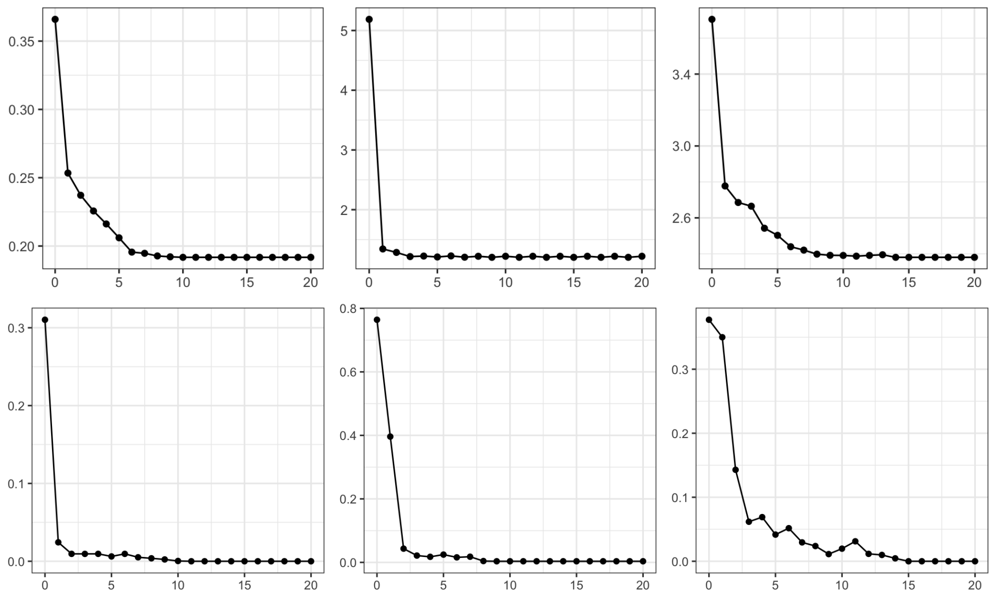

Figure 4: Amplitude prediction errors, as defined by (1) (top row), and relative changes, as defined by (2) (bottom row), in a single simulation run under three simulation scenarios: bimodal (left column), B-spline with independent amplitude and phase (middle column), and B-spline with dependent amplitude and phase (right column). The x-axis denotes the iteration number.

$(q_0,\gamma_0)(u)\}\{(q_j,\gamma_j)(u)-(q_0,\gamma_0)(u)\}]\mathrm{d}u$, which then equals

$$\int_0^1 \mathrm{E}\big[\{(q_i,\gamma_i)(u)-\mu_q(u)+\mu_q(u)-(q_0,\gamma_0)(u)\}\{(q_j,\gamma_j)(u)-\mu_q(u)+\mu_q(u)-(q_0,\gamma_0)(u)\}\big]\mathrm{d}u$$
$$=\int_0^1 \big[\mathrm{cov}\{(q_i,\gamma_i)(u),(q_j,\gamma_j)(u)\}-\mathrm{cov}\{(q_i,\gamma_i)(u),(q_0,\gamma_0)(u)\}$$
$$-\mathrm{cov}\{(q_0,\gamma_0)(u),(q_j,\gamma_j)(u)\}+\mathrm{var}\{(q_0,\gamma_0)(u)\}\big]\mathrm{d}u$$
$$=\int_0^1 \big[\mathrm{var}\{(q_0,\gamma_0)(u)\}-\mathrm{cov}\{(q_0,\gamma_0)(u),(q_j,\gamma_j)(u)\}\big]\mathrm{d}u$$
$$+\int_0^1 \big[\mathrm{var}\{(q_0,\gamma_0)(u)\}-\mathrm{cov}\{(q_0,\gamma_0)(u),(q_i,\gamma_i)(u)\}\big]\mathrm{d}u$$
$$-\int_0^1 [\mathrm{var}\{(q_0,\gamma_0)(u)\}-\mathrm{cov}\{(q_i,\gamma_i)(u),(q_j,\gamma_j)(u)\}]\mathrm{d}u = V_a(h_{0j})+V_a(h_{i0})-V_a(h_{ij}), \tag{4}$$

where $h_{ij}=\|s_i-s_j\|_2$, for $i,j=1,\dots,n$. The last equality results from the definition of the amplitude trace-variogram, which by Fubini's theorem and stationarity assumption where $\mathrm{var}\{(q_s,\gamma_s)(t)\}=\sigma^2(t)$ does not depend on the spatial location $s\in\mathcal{D}$ implies that

$$V_a(h_{ij})=\frac{1}{2}\mathrm{E}(\|(q_i,\gamma_i)-(q_j,\gamma_j)\|^2)=\int_0^1 \big[\mathrm{var}\{(q_0,\gamma_0)(u)\}-\mathrm{cov}\{(q_i,\gamma_i)(u),(q_j,\gamma_j)(u)\}\big]\mathrm{d}u.$$

Plugging (4) into (3) results in $\eta^T\mathcal{V}_a\eta$, where $\mathcal{V}_a=[V_a(h_{0j})+V_a(h_{i0})-V_a(h_{ij})]_{n\times n}$. □

The proof of Proposition 2 follows along almost identical lines. The changes are to replace the amplitude components in the proof of Proposition 1 by phase components, and to condition on the shape random field $\mathcal{S}$ in the expectation. The expected phase prediction error given $\mathcal{S}$ is

$$\mathrm{E}(\|\tilde{\psi}_0-\psi_0\|^2\mid\mathcal{S})=\sum_{i=1}^n\sum_{j=1}^n\zeta_i\zeta_j\int_0^1 \mathrm{E}[\{\psi_i(u)-\psi_0(u)\}\{\psi_j(u)-\psi_0(u)\}\mid\mathcal{S}]\mathrm{d}u. \tag{5}$$

Under the assumptions of the Proposition, we have $E(\psi_i(t) \mid \mathcal{S}) = \psi(t)$ and $\mathrm{var}(\psi_i(t) \mid \mathcal{S})$ are constant in space for any t. Then,

$$\begin{aligned}
&\int_0^1 \mathrm{E}[\{\psi_i(u) - \psi_0(u)\}\{\psi_j(u) - \psi_0(u)\} \mid \mathcal{S}]\mathrm{d}u \\
&= \int_0^1 \mathrm{E}[\{\psi_i(u) - \psi(u) + \psi(u) - \psi_0(u)\}\{\psi_j(u) - \psi(u) + \psi(u) - \psi_0(u)\} \mid \mathcal{S}]\mathrm{d}u \\
&= \int_0^1 \mathrm{cov}\{\psi_i(u), \psi_j(u) \mid \mathcal{S}\} - \mathrm{cov}\{\psi_i(u), \psi_0(u) \mid \mathcal{S}\} - \mathrm{cov}\{\psi_0(u), \psi_j(u) \mid \mathcal{S}\} + \mathrm{var}\{\psi_0(u) \mid \mathcal{S}\}\mathrm{d}u \\
&= \int_0^1 \mathrm{var}\{\psi_0(u) \mid \mathcal{S}\} - \mathrm{cov}\{\psi_0(u), \psi_j(u) \mid \mathcal{S}\}\mathrm{d}u + \int_0^1 \mathrm{var}\{\psi_0(u) \mid \mathcal{S}\} - \mathrm{cov}\{\psi_0(u), \psi_i(u) \mid \mathcal{S}\}\mathrm{d}u \\
&- \int_0^1 \mathrm{var}\{\psi_0(u) \mid \mathcal{S}\} - \mathrm{cov}\{\psi_i(u), \psi_j(u) \mid \mathcal{S}\}\mathrm{d}u = V_p(h_{0j,\omega}) + V_p(h_{i0,\omega}) - V_p(h_{ij,\omega}),
\end{aligned} \tag{6}$$

where $h_{ij,\omega} = \sqrt{\|s_i - s_j\|_2^2 + \omega \cdot d_{sh}(q_i, q_j)^2}$ $(i = 0, 1, \dots, n;\ j = 0, 1, \dots, n)$ and we use the property that for random variables X, Y, Z, $\mathrm{cov}(X, Y|Z) = \mathrm{E}(XY|Z) - \mathrm{E}(X|Z)\mathrm{E}(Y|Z)$. □

Appendix C Consistent alignment and convergence of amplitude kriging estimator under one-dimensional model

In Section 2.2 in the main article, we describe an approach for template-based alignment of independent functional data. A model-based formulation of the alignment procedure considers the model $q_i(t) = \{(c_i\mu_q + e_i)\circ\gamma_i^{-1}\}(t)\left\{\dot{\gamma}_i^{-1}(t)\right\}^{1/2}$, $t \in [0, 1]$, $i = 1, \dots, n$ for functional observations in $\mathcal{Q}$, where μ_q is a template function, $c_i \in R_+$ and $e_i(t)$ are realizations of an error process $e(t)$. Unfortunately, it is well-known that the template μ_q is not identifiable except when $e(t) \equiv 0$; in other words, μ_q can only be estimated consistently (as $n \to \infty$) under a one-dimensional model where randomness arises purely through scale variation (Kurtek and Srivastava, 2011). This is not an artifact of the square-root slope transform framework, but rather relates to the fact that e_i and γ_i cannot be disentangled in general when e_i is infinite-dimensional; see Chakraborty and Panaretos (2021) for a different perspective that leads to the same conclusion. Thus, under a general functional model that is not one-dimensional, the warping functions γ_i, $i = 1, \dots, n$ cannot be consistently estimated. An important implication of this is that, in the spatial setting, it is also generally not be possible to consistently estimate the warping functions $\{\gamma_{s_i},\ s_i \in \mathcal{D}\}$ $(i = 1, \dots, n)$, and as a result, to construct consistent estimators of the amplitude and phase trace-variograms.

In the spatial setting, in order to decompose the trace-variogram into amplitude and phase components, consider the model

$$q_{s_i}(t) = (\mu_q + e_{s_i}, \gamma_{s_i}^{-1})(t) = \left\{(\mu_q + e_{s_i})\circ\gamma_{s_i}^{-1}\right\}(t)\left\{\dot{\gamma}_{s_i}^{-1}(t)\right\}^{1/2},\ t \in [0, 1],\ s_i \in \mathcal{D},\ i = 1, \dots, n \tag{7}$$

for the observed data with unobserved μ_q and γ_{s_i}. Equivalently, $(q_{s_i}, \gamma_{s_i})(t) = \mu_q(t) + e_{s_i}(t)$. We assume that the data is generated from two functional random fields: the *amplitude random field* $\{(q_s, \gamma_s),\ s \in \mathcal{D}\}$, and the corresponding *phase random field* $\{\psi_s = \dot{\gamma}_s^{1/2},\ s \in \mathcal{D}\}$, associated with the functional random field $\{q_s,\ s \in \mathcal{D}\}$. The $\mu_q \in \mathcal{Q}$ is a deterministic mean amplitude, constant in space, and $\{e_s\}$ and $\{\psi_s\}$ are independent functional random fields assuming values in $\mathcal{Q}$ and Ψ, respectively, with $\mathrm{E}(e_s) \equiv 0\ \forall\ s \in \mathcal{D}$. The random field $\{\psi_s\}$ depends on $\{q_s\}$ only through the mean μ_q. Under the model, the amplitude of q_{s_i} is (q_{s_i}, γ_{s_i}), free of phase variation, and μ_q is thus the common mean amplitude. Consistent estimation of μ_q for alignment of spatial functional data under (7) is again unattainable for the same reasons as in the independent case. Instead, one can show that, under the simplified one-dimensional model, the amplitude kriging estimator $\tilde{q}_0$, at spatial location $s_0 \in \mathcal{D}$, converges in L^2 to an element of the true orbit $[q_0]$. This leads to the following proposition.

Proposition 3. *Consider observations $q_{s_1}, \dots, q_{s_n}$ and the function to predict q_{s_0} from the one-dimensional model*

$$q_s = (c_s\mu_q, \gamma_s^{-1}),$$

where $\{c_s\}$ is a scalar, positive, isotropic random field on $\mathcal{D}$, $\mu_q \in \mathcal{Q}$ is a deterministic mean function, and $\gamma_s \in \Gamma$ is a random warping function. Suppose the variogram of $\{c_s\}$ is continuous in a neighborhood of 0 and s_0 is a limit point of $\{s_1, \dots, s_n\}$ as $n \to \infty$. Then, the estimator $\tilde{q}_{s_0}$ converges in L^2 to an element of the orbit $[q_{s_0}]$ as $n \to \infty$.

Proof. We simplify the notation by referring to $s_i,\ i = 0, 1, \dots, n$ as $i = 0, 1, \dots, n$, i.e., $q_i = q_{s_i},\ c_i = c_{s_i}, \gamma_i = \gamma_{s_i}$. The proof is based on (i) establishing that minimizing the amplitude prediction error $E(\|\tilde{q}_0 - (q_0, \gamma_0)\|^2)$ to obtain the coefficient vector η can be reduced to solving the same problem for scalars $c_1, \dots, c_n$ pertaining to the scalar random field $\{c_s\}$, and (ii) using known results on consistency of the kriging estimate based on $c_1, \dots, c_n$ to thus establish the required result.

Since μ_q is unknown, we choose q_1 as a template to align the rest of the data and obtain their phase components; we will see that this choice has no bearing on the result. Under the one-dimensional model, it is possible to recover the phase functions $\hat{\gamma}_i,\ i = 2, \dots, n$ in terms of γ_1 regardless of what μ_q is:

$$\begin{aligned}\hat{\gamma}_i =& \arg\min_{\gamma} \|(q_i, \gamma) - q_1\|^2 = \arg\min_{\gamma} \{\|(q_i, \gamma)\|^2 - 2\langle (q_i, \gamma), q_1\rangle + \|q_1\|^2\} \\ =& \arg\min_{\gamma} \{\|q_i\|^2 - 2\langle (q_i, \gamma), q_1\rangle + \|q_1\|^2\} = \arg\max_{\gamma} \langle (q_i, \gamma), q_1\rangle \\ =& \arg\max_{\gamma} c_1 c_i \langle (\mu_q, \gamma_i^{-1} \circ \gamma), (\mu_q, \gamma_1^{-1})\rangle = \gamma_i \circ \gamma_1^{-1},\end{aligned}$$

where the third equality again uses the norm-preserving action of Γ on $\mathcal{Q}$. The key observation is that in the one-dimensional model, optimal alignment under the norm-preserving action is independent of the scales of the functions.

The aligned functions (with respect to q_1 as the template) are then given by $(q_i, \hat{\gamma}_i) = c_i(\mu_q, \gamma_1^{-1}),\ i = 2, \dots, n$. Indeed, $\hat{\gamma}_1$ is then the identity map. In order to compute the prediction error, we require an estimate of γ_0. Since any estimate of γ_0 will not depend on the scale c_0 of q_0, we choose $\hat{\gamma}_0 = \gamma_0 \circ \gamma_1^{-1}$.

The next key observation is that despite not knowing q_0, it is true that $(q_0, \hat{\gamma}_0) \in [q_0]$ regardless of what $\hat{\gamma}_0$ is, and it thus suffices to show that $\tilde{q}_0 \to (q_0, \hat{\gamma}_0)$ in L^2 as $n \to \infty$. Using the aligned functions $\{(q_i, \hat{\gamma}_i)\}$ and the estimate $\hat{\gamma}_0$, note that the coefficient η that defines the estimator $\tilde{q}_0$ is obtained by minimizing the amplitude prediction error $\mathrm{E}(\|\sum_{i=1}^{n} \eta_i (q_i, \hat{\gamma}_i) - (q_0, \hat{\gamma}_0)\|^2)$, which reduces to

$$\begin{aligned}&\mathrm{E}\Big(\|(c_0 - \sum_{i=1}^{n} \eta_i c_i)(\mu_q, \gamma_1^{-1})\|^2\Big) = \mathrm{E}\Big((c_0 - \sum_{i=1}^{n} \eta_i c_i)^2 \|(\mu_q, \gamma_1^{-1})\|^2\Big) = \mathrm{E}\Big((c_0 - \sum_{i=1}^{n} \eta_i c_i)^2 \|\mu_q\|^2\Big) \\ =& \mathrm{E}\Big((c_0 - \sum_{i=1}^{n} \eta_i c_i)^2\Big) \|\mu_q\|^2.\end{aligned}$$

Thus, after eliminating the phase variation in the one-dimensional model, amplitude kriging reduces to univariate kriging of the scaling terms since $\|\mu_q\|^2 < \infty$. We also observe that we could have chosen any of the $q_i,\ i = 2, \dots, n$ as a template.

We now examine consistency of $\hat{c}_0 = \sum_{i=1}^{n} \hat{\eta}_i c_i$, where $\hat{\eta}_i$ is obtained by minimizing the amplitude prediction error. When the variogram of $\{c_s\}$ is known and continuous in a neighborhood of 0, and a limit point of $\{s_1, \dots, s_n\}$ is s_0 as $n \to \infty$, the expected prediction error $\mathrm{E}\{(\hat{c}_0 - c_0)^2\} \to 0$ as $n \to \infty$ (Yakowitz and Szidarovszky, 1985). When the variogram is unknown, it can be replaced by the empirical amplitude trace-variogram

$$\begin{aligned}\hat{V}_a(h) =& \frac{1}{2|N(h)|} \sum_{i,j \in N(h)} \|(q_i, \hat{\gamma}_i) - (q_j, \hat{\gamma}_j)\|^2 = \frac{1}{2|N(h)|} \sum_{i,j \in N(h)} \|(c_i \mu_q, \gamma_1) - (c_j \mu_q, \gamma_1)\|^2 \\ =& \frac{1}{2|N(h)|} \sum_{i,j \in N(h)} \|c_i \mu_q - c_j \mu_q\|^2 = \|\mu_q\|^2 \cdot \hat{V}_c(h),\end{aligned}$$

where $\hat{V}_c(h) = \frac{1}{2|N(h)|} \sum_{i,j \in N(h)} \|c_i - c_j\|^2$, and we have again used the fact that under the one-dimensional model $\hat{\gamma}_i = \gamma_i \circ \gamma_1^{-1}$ when q_1 is used as the template. If observed locations are regularly spaced on a grid with fixed spacing but increasing domain, Davis and Borgman (1982) proved that the empirical variogram $\hat{V}_c(h)$ is a consistent estimator

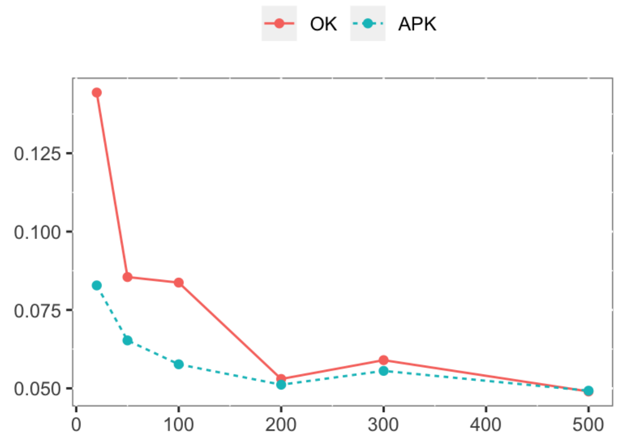

Figure 5: Convergence of prediction error for ordinary kriging (OK, red) and amplitude-phase kriging (APK, green) as sample size increases. The x-axis is the sample size and the y-axis is the median amplitude prediction error across 50 repetitions.

for the true variogram, provided that the random field $\{c_s,\ s \in \mathcal{D}\}$ is m-dependent; m-dependence is a condition that ensures that elements in a random field with large enough distance are independent.

Under mild assumptions, the consistent empirical variogram enables consistent estimators of parameters in parametric variogram models, which are obtained by the least squares method (Lahiri, Lee and Cressie, 2002). Furthermore, Yakowitz and Szidarovszky (1985) and Stein (1988) discuss the effects of misspecification of the variogram on kriging. Given the true covariance function C, the best linear unbiased estimator is $\hat{c}_0$. If the covariance function is misspecified as C^*, we have the best estimator $\hat{c}_0^*$. Stein (1988) demonstrates that if C^* and C are compatible (Ibragimov and Rozanov, 2012), then

$$\frac{\mathrm{E}\big((\hat{c}_0 - c_0)^2\big)}{\mathrm{E}\big((\hat{c}_0^* - c_0)^2\big)} \to 1, \quad \frac{\mathrm{E}\big((\hat{c}_0 - c_0)^2\big)}{\mathrm{E}^*\big((\hat{c}_0^* - c_0)^2\big)} \to 1, \quad n \to \infty,$$

where E^* is the expectation under the misspecified covariance. Since the prediction error $\mathrm{E}((\hat{c}_0 - c_0)^2) \to 0$ as $n \to \infty$ given the true variogram of $\{c_i\}$, the prediction error when using C^* also converges to 0. $\square$

Proposition 3 further guarantees convergence of Algorithm 1, as specified in Section 4.1 in the main article, for the one-dimensional model. This is due to the fact that the spatial dependency in the data arises through the scaling coefficients only, and the computation of the weights η at each iteration is not dependent on $\{\hat{\gamma}_i\}$.

We report results of a simulation that validates Proposition 3. We generate n spatial functional observations from the one-dimensional model on the spatial domain $\mathcal{D} = [0,1]^2$. The spatial locations are drawn uniformly. We set $f(t) = \exp[-(t-2)^2/(2{\times}1.5^2)]{+}\exp[-(t+2)^2/(2{\times}1.5^2)]$, $t \in [-5,5]$. The vector of scaling coefficients, $[c_1,\dots,c_n]$, is sampled from a multivariate normal distribution with mean vector $(5,\dots,5)^T$ and Matérn covariance with scale parameter 1, smoothing parameter 0.5, and range $\ell = 0.5$; the error constants, $[e_1,\dots,e_n]$, are sampled from a multivariate normal distribution with mean $(5,\dots,5)^T$ and Matérn covariance with scale parameter 1, smoothing parameter 0.5 and range $\ell = 0.5$. The warping functions $\gamma_i,\ i = 1,\dots,n$ are generated using a Beta CDF with correlated uniform parameters as in the main manuscript; the parameters are chosen as $B = 1.5$ and $\ell = 0.5$. For different sample sizes n, we simulate data in this fashion 50 times and focus on prediction at site $(0.5, 0.5)$ via ordinary kriging and amplitude-phase kriging. For each sample size, we compute the median of the amplitude error $d_a(\hat{q}_0, q_0)/\|q_0\|$ ($\hat{q}_0$ and q_0 are the SRSFs of the predicted and true functions, respectively), across the 50 repetitions, to assess consistency of amplitude prediction based on these two methods. The results are presented in Figure 5. As expected, the error associated with the amplitude-phase kriging predictions is much smaller than the one associated with the ordinary kriging predictions when sample size is small. As sample size increases, the two methods perform similarly and converge to a very small error value. This is expected since both approaches yield consistent estimators. We note that the final error is not 0 due to numerical approximations in the two procedures. In many real data scenarios,

Table 1
Average prediction errors (SD) using metrics $E1$-$E5$, across 50 different replicates, for amplitude-phase kriging (APK), two-stage kriging (TSK), ordinary kriging (OK), and universal kriging (UK), when sites are randomly sampled on the spatial domain. $E2$ is divied by 100 and $E4$ is multiplied by 10 to adjust the scale. B controls the magnitude of phase variation and ℓ is the range parameter of spatial correlation for amplitude and phase.

				Bimodal			
B	ℓ	Method	E1	E2	E3	E4	E5
0.5	$8^{1/2}$	APK	0.99 (0.35)	**0.42 (0.15)**	**0.49 (0.13)**	**0.13 (0.04)**	8.22 (5.8)
		TSK	**0.96 (0.34)**	**0.42 (0.14)**	**0.49 (0.13)**	0.16 (0.06)	11.78 (7.81)
		OK	1.48 (0.79)	0.62 (0.27)	0.74 (0.31)	0.14 (0.05)	**5.58 (2.39)**
		UK	4.11 (6.91)	3.3 (9.46)	1.55 (2.02)	0.19 (0.11)	9.88 (8.3)
	$18^{1/2}$	APK	0.88 (0.36)	0.37 (0.13)	**0.44 (0.1)**	**0.1 (0.03)**	5.65 (3.85)
		TSK	**0.85 (0.36)**	**0.36 (0.13)**	**0.44 (0.11)**	0.14 (0.05)	10.46 (6.68)
		OK	1.2 (0.65)	0.51 (0.23)	0.6 (0.2)	0.12 (0.04)	**4.36 (2.7)**
		UK	2.07 (2.25)	1.01 (1.6)	1.03 (1.1)	0.16 (0.08)	6.6 (4.75)
1	$8^{1/2}$	APK	1.26 (0.48)	1.36 (1.6)	**0.74 (0.25)**	**0.49 (0.15)**	26.08 (11.2)
		TSK	**1.23 (0.49)**	**1.31 (1.53)**	0.75 (0.24)	0.5 (0.14)	29.86 (12.44)
		OK	6.75 (4.03)	3.59 (1.89)	3.59 (2.03)	0.69 (0.29)	**16.97 (7.52)**
		UK	6.66 (3.41)	4.46 (5.78)	3.32 (1.57)	0.7 (0.23)	18.38 (6.7)
	$18^{1/2}$	APK	1.11 (0.41)	1.3 (1.48)	**0.68 (0.24)**	**0.46 (0.15)**	27.43 (13.78)
		TSK	**1.09 (0.4)**	**1.27 (1.44)**	0.69 (0.24)	**0.46 (0.15)**	29.33 (14.44)
		OK	5.99 (3.55)	3.34 (2.1)	3.09 (1.4)	0.62 (0.23)	**16.53 (8.45)**
		UK	5.59 (2.58)	3.18 (1.91)	2.94 (1.1)	0.65 (0.23)	16.61 (7.88)
				B-spline Scenario 1 (independent)			
B	ℓ	Method	E1	E2	E3	E4	E5
0.5	$8^{1/2}$	APK	1.2 (0.45)	2.08 (1.67)	**1.93 (0.36)**	**0.86 (0.18)**	2.43 (0.76)
		TSK	1.2 (0.37)	2.12 (1.67)	2.06 (0.4)	1.1 (0.25)	3.05 (0.79)
		OK	**0.9 (0.17)**	**2 (1.45)**	2.02 (0.42)	0.95 (0.16)	**1.44 (0.3)**
		UK	1.19 (0.29)	2.34 (1.7)	2.41 (0.55)	0.9 (0.25)	1.83 (0.42)
	$18^{1/2}$	APK	1.05 (0.29)	1.92 (0.94)	**1.84 (0.36)**	0.85 (0.29)	1.90 (0.46)
		TSK	1.07 (0.33)	1.90 (0.90)	1.86 (0.35)	0.95 (0.36)	2.27 (0.60)
		OK	**0.83 (0.14)**	**1.89 (0.95)**	1.92 (0.40)	**0.80 (0.25)**	**1.34 (0.33)**
		UK	0.93 (0.17)	1.97 (0.93)	2.06 (0.45)	0.84 (0.26)	1.47 (0.31)
1	$8^{1/2}$	APK	1.31 (0.39)	2.49 (1.46)	**2.17 (0.49)**	**1.09 (0.17)**	3.53 (0.83)
		TSK	1.33 (0.45)	**2.48 (1.43)**	2.33 (0.46)	1.38 (0.28)	4.14 (0.91)
		OK	**1.2 (0.36)**	2.73 (1.46)	2.61 (0.63)	1.1 (0.17)	**2.28 (0.76)**
		UK	1.89 (1.8)	4.47 (7.92)	3.22 (1.26)	1.26 (0.3)	3.2 (2.77)
	$18^{1/2}$	APK	1.12 (0.28)	2.6 (1.86)	**2.03 (0.46)**	**1.04 (0.29)**	2.83 (0.91)
		TSK	1.15 (0.34)	**2.57 (1.76)**	2.08 (0.5)	1.2 (0.4)	3.3 (1.17)
		OK	**1.09 (0.26)**	2.9 (1.8)	2.52 (0.63)	1.17 (0.26)	**2.15 (0.72)**
		UK	1.17 (0.28)	2.92 (1.66)	2.65 (0.7)	1.22 (0.28)	2.26 (0.68)

the number of observations available for kriging is limited. Thus, lower amplitude errors and faster convergence of the error, with respect to sample size, of amplitude-phase kriging as compared to ordinary kriging in Figure 5 clearly summarizes the benefits of the proposed approach in the case of the one-dimensional model.

Appendix D Invariance of spatial hierarchical clustering to global scaling of variogram

The proposed amplitude-phase hierarchical clustering approach is invariant to a global scaling of the amplitude and phase trace-variograms, since hierarchical clustering, in general, is invariant to the scale of the dissimilarity matrix. To show this, given observations $\{q_i,\ s_i \in \mathcal{D}\}$ $(i = 1, \dots, n)$, let us consider two new dissimilarity measures, based on those defined in (14) in the main manuscript:

$$d^*_{A,ij} = d_a(q_i, q_j) \times CV_a(\|s_i - s_j\|),\ \ d^*_{P,ij} = d_p(q_i, q_j) \times CV_p(h_{ij,\omega}),\ \ i, j = 1, \dots, n, \tag{8}$$

where $C \in R_+$ is a scaling constant. In hierarchical clustering, the elemental procedure merges two sets of objects that have the smallest distance. The definition of distance between two sets is determined by a linkage function, e.g., complete or average linkage. Taking complete linkage as an example, for two sets of observations $S_1,\ S_2$, the new spatially weighted amplitude distance between them is $\max\{d^*_{A,ij}\ :\ i \in S_1,\ j \in S_2\} = C \max\{d_{A,ij}\ :\ i \in S_1,\ j \in$

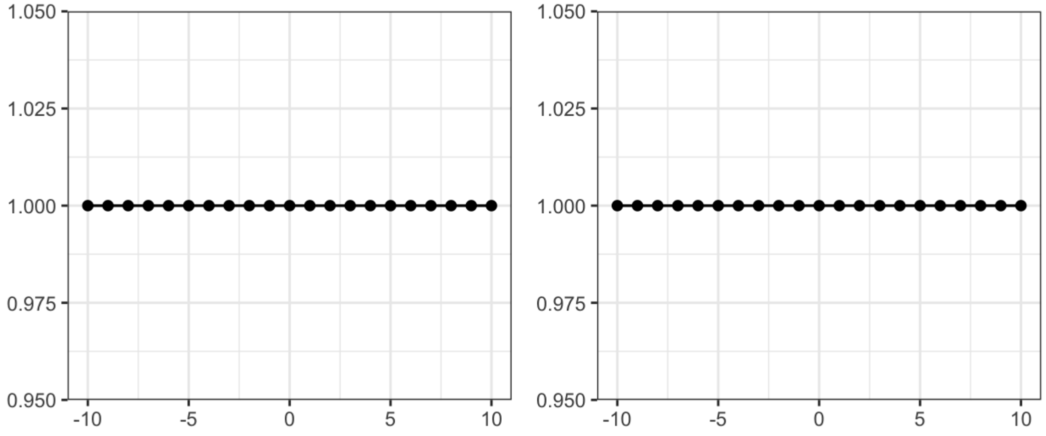

Figure 6: Rand indices between estimated clusters when $C = 1$ and estimated for different values of C. The x-axis is $\log_2(C)$. The y-axis is the rand index. The left and right panels consider amplitude and phase clustering, respectively.

$S_2\}$, where $d_{A,ij} = d_a(q_i, q_j) \times V_a(\|s_i - s_j\|)$ is the dissimilarity when $C = 1$ as in (14) in the main manuscript. The same holds for the spatially weighted phase distance between the two sets of observations. Thus, regardless of the value of C, one always merges the two sets observations with the smallest distance $\max\{d_{A,ij} : i \in S_1, j \in S_2\}$. While this example used complete linkage, the same holds for other linkage functions.

To show that this is true empirically, we generated simulated data using the model described in Section 6.2 in the main manuscript, and conducted amplitude-phase clustering using the new dissimilarities in (8) for $C = 2^m$, $m = -10, -9, \dots, 10$. Choosing the estimated amplitude and phase partitions under $C = 1$ as the baseline, we computed the rand index between estimated partitions for each value of C as indicated above, and the baseline estimated partitions. Figure 6 shows that the estimated amplitude and phase clusters do not depend on the value of C since all of the rand indices are exactly 1 for both amplitude and phase, i.e., the clustering results are identical for each value of C.

Appendix E Implementation and additional results

Estimation of phase via regularized alignment. When we estimate the phase component of each function for phase kriging, we utilize a regularized alignment method (Srivastava and Klassen, 2016) to increase robustness to noise or large shape variation in the observed spatial functional data. The optimal warping function that aligns q_2 to q_1 is estimated using:

$$\gamma^* = \underset{\gamma \in \Gamma}{\arg\min} \{\|q_1 - (q_2, \gamma)\|^2 + \lambda \|\psi - \psi_{id}\|^2\}, \tag{9}$$

where $\psi = \dot{\gamma}^{1/2}$ is the square-root slope transformed γ and $\psi_{id}(t) = 1$ is the square-root slope transformed identity warping $\gamma_{id}(t) = t$. The tuning parameter λ is selected in each run of leave-one-out cross-validation by additional five-fold cross-validation. Specifically, the leave-one-out cross-validation training set is randomly divided into five folds. Given a candidate λ, we use each fold as the prediction target, and use the data in the other four folds to realize the phase kriging prediction. We compute the sum of squared extrinsic phase distances (Definition 2 in the main article) between the predicted and true phase functions. We repeat the five-fold cross-validation ten times and select the optimal λ as the one that minimizes the average prediction error. Despite the complex hierarchical cross-validation structure of phase kriging, the computation is efficient in practice.

Additional kriging simulation results. We evaluate our approach across different spatial range parameters ℓ in the amplitude and phase spatial covariances (set to be equal across the two components). We perform the same simulation as in Section 7.1 in the main article, but we only consider the bimodal setting and the B-spline setting with independent shape and phase components. We again consider different scales for the phase variation, which are controlled by the parameter B. The prediction results are reported in Table 1. We report the mean and standard deviation of error metrics $E1$-$E5$ for the four competing methods: amplitude-phase kriging (APK), two-stage kriging (TSK), ordinary kriging without alignment (OK), and universal kriging without alignment (UK). Best performance is highlighted in bold. An

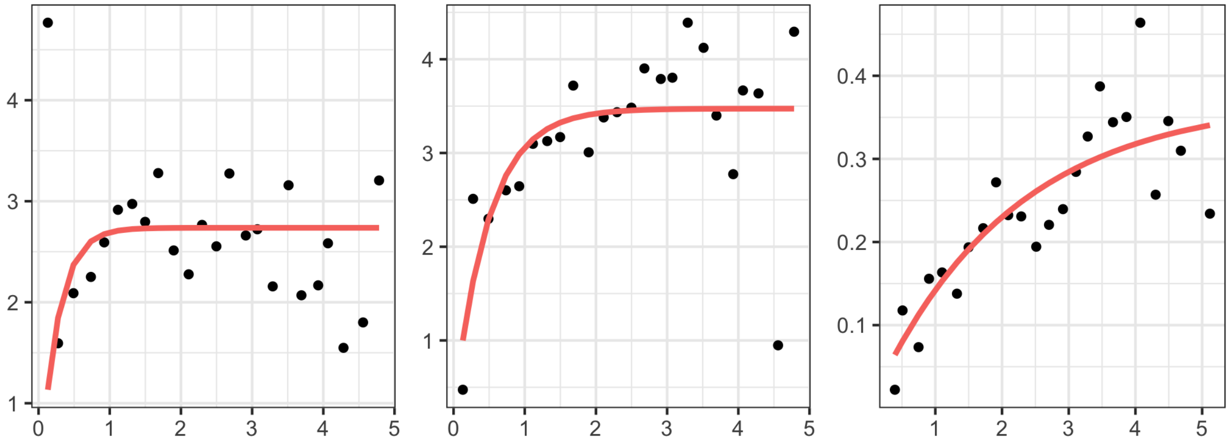

Figure 7: Estimation of trace-variograms for prediction at site 1 under the B-spline Scenario 1 simulation with $B = 1$; site 1 was left out and the rest of the observations were used to estimate the trace-variograms. The dots represent the empirical L^2 (ordinary kriging), amplitude and phase trace-variograms. Estimates of the trace-variograms (red curves) were obtained by fitting a Matérn variogram model to the empirical trace-variograms.

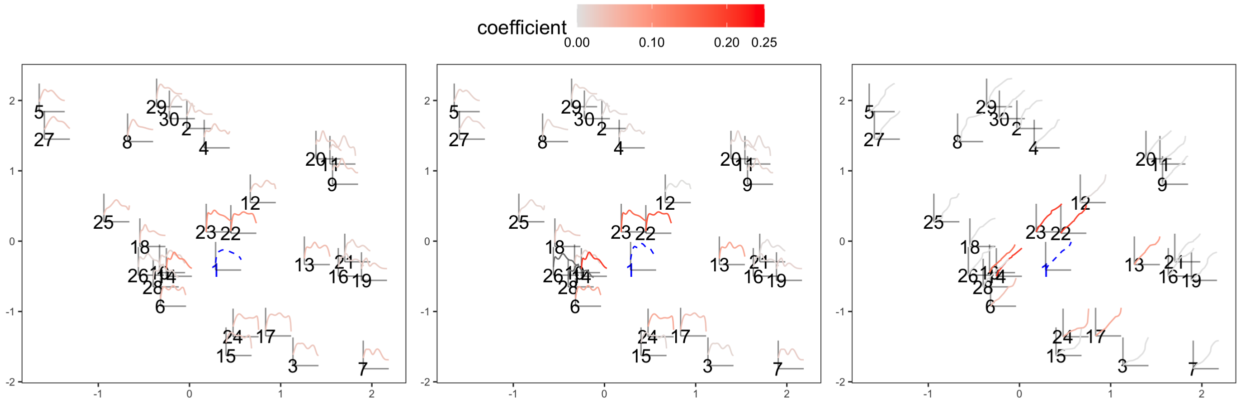

Figure 8: Ordinary (left), amplitude (middle) and phase (right) kriging maps for prediction at site 1 under the B-spline Scenario 1 simulation with $B = 1$. The magnitude of kriging coefficients to construct the estimators (dashed blue) are shown in red.

additional finding based on this simulation is that stronger spatial dependency enhances prediction performance of all methods, as expected, but generally does not change their relative performance. In Figures 7 and 8, we also display an example of the empirical and fitted trace-variograms, and the corresponding values of kriging coefficients for the B-spline Scenario 1 simulation. The kriging maps in Figure 8 illustrate the contribution of each observed function to the kriging estimators. The results suggest that ordinary kriging tends to assign non-zero coefficients to more of the observed functions, resulting in a prediction with fewer distinguishable amplitude features. On the other hand, the amplitude and phase predictions are dominated by the observations at sites that are spatially close to site 1.

Sensitivity to different levels of pre-smoothing. We perform the same leave-one-out cross-validation on the 24 daily average ozone functions as in Section 8.1 in the main article, but use three different levels of spline-based smoothing: $\iota = 1 \times 10^{-4}$ (low), $\iota = 3 \times 10^{-4}$ (medium; results also reported in the main article) and $\iota = 5 \times 10^{-4}$ (high). We report the mean of the five error metrics, $E1$-$E5$, for each level of smoothing in Table 2. When the input data is relatively smooth ($\iota = 5 \times 10^{-4}$), the difference in amplitude errors between the proposed method and ordinary kriging is very small; two-stage kriging results in the largest errors. This is due to the smoothing procedure, which smooths out notable shape characteristics of the functions. This is beneficial for ordinary kriging that generally underestimates prominent shape features in predicted functions due to misalignment of the observed data. On the other hand, the

Table 2
Leave-one-out cross-validation average prediction errors of amplitude-phase kriging (APK), two-stage kriging (TSK), and ordinary kriging (OK) for the smoothed ozone data in North California. All numbers were multiplied by 1000.

ι	Method	E1	E2	E3	E4	E5
1×10^{-4}	APK	4.68	**16.32**	3.41	70.31	6.89
	TSK	**4.34**	16.52	**3.36**	**65.91**	**6.54**
	OK	4.83	18.25	3.98	76.69	6.67
3×10^{-4}	APK	4.88	**7.57**	**1.94**	49.02	7.17
	TSK	4.67	8.79	2.21	**45.9**	6.6
	OK	**4.17**	8.01	2.23	53.64	**6.44**
5×10^{-4}	APK	**4.03**	6.93	**1.62**	**44.88**	6.34
	TSK	4.55	8.32	2.17	63.78	6.74
	OK	4.09	**6.43**	**1.62**	51.21	**6.32**

performance of two-stage kriging suffers in this case, because there are fewer prominent features that can be used to inform the global alignment. In contrast, when less smoothing is applied to the observed functions ($\iota = 1 \times 10^{-4}$), the amplitude prediction errors are much smaller for the proposed method as compared to ordinary kriging; the amplitude mean squared error ($E3$) is reduced by 14% compared to ordinary kriging. At the same time, the proposed approach has similar prediction accuracy as two-stage kriging in terms of amplitude. This is due to the fact that more shape characteristics of the observed functions are preserved after smoothing; further, the proposed approach is much more effective in capturing these features in the amplitude predictions than ordinary kriging.

In terms of phase, the proposed method outperforms ordinary kriging for all values of the smoothing parameter; the reduction in the phase mean squared error ($E4$) ranges from 8% to 12%. Amplitude-phase kriging significantly outperforms two-stage kriging in terms of phase prediction when a considerable amount of smoothing is applied to the data. On the other hand, two-stage kriging outperforms amplitude-phase kriging for the two lower values of the smoothing parameter. Overall, it is evident that amplitude-phase kriging is fairly robust under different magnitudes of smoothing of the observed functions.

Canadian weather data clustering without spatial dependency. The clustering techniques discussed in the main article account for the spatial dependency across functional observations. The spatial dependency is encoded in the dissimilarity matrix via weights, where the discrepancy between pairs of functions near each other, with respect to the distance on the domain, is down-weighted. The weight, computed using the fitted trace-variogram, works as an empirical prior, where the dependency decays as the domain distance increases, and its range and rate of decay depend on the data. In particular, if the data suggest no spatial dependency, spatially-weighted clustering is the same as standard hierarchical clustering without spatial information. Spatial weighting tends to preserve connectivity of clusters and often results in more interpretable results.

To show the difference between clustering with and without spatial adjustment, we applied standard hierarchical clustering, with average linkage, to the same data as considered in Section 8.2 in the main article. The results are shown in Figure 9, and are directly comparable to Figure 3 in the main article. The partitions estimated using hierarchical clustering without accounting for spatial dependency tend to be more scattered, e.g., cluster 2 (yellow) in amplitude and phase clustering. Such results are difficult to interpret and relate to geological factors. In addition, standard hierarchical clustering of this data results in many more singleton clusters.

References

Chakraborty, A., Panaretos, V., 2021. Functional registration and local variations: Identifiability, rank, and tuning. Bernoulli 27, 1103–1130.

Davis, B.M., Borgman, L.E., 1982. A note on the asymptotic distribution of the sample variogram. Journal of the International Association for Mathematical Geology 14, 189–193.

Ibragimov, I.A., Rozanov, Y.A., 2012. Gaussian Random Processes. volume 9. Springer Science & Business Media.

Kurtek, S., Srivastava, A., 2011. Signal estimation under random time-warpings and nonlinear signal alignment, in: Advances in Neural Information Processing Systems, pp. 676–683.

Lahiri, S.N., Lee, Y., Cressie, N., 2002. On asymptotic distribution and asymptotic efficiency of least squares estimators of spatial variogram parameters. Journal of Statistical Planning and Inference 103, 65–85.

Srivastava, A., Klassen, E.P., 2016. Functional and Shape Data Analysis. volume 475. Springer.

Stein, M.L., 1988. Asymptotically efficient prediction of a random field with a misspecified covariance function. Annals of Statistics , 55–63.

Yakowitz, S., Szidarovszky, F., 1985. A comparison of kriging with nonparametric regression methods. Journal of Multivariate Analysis 16, 21–53.

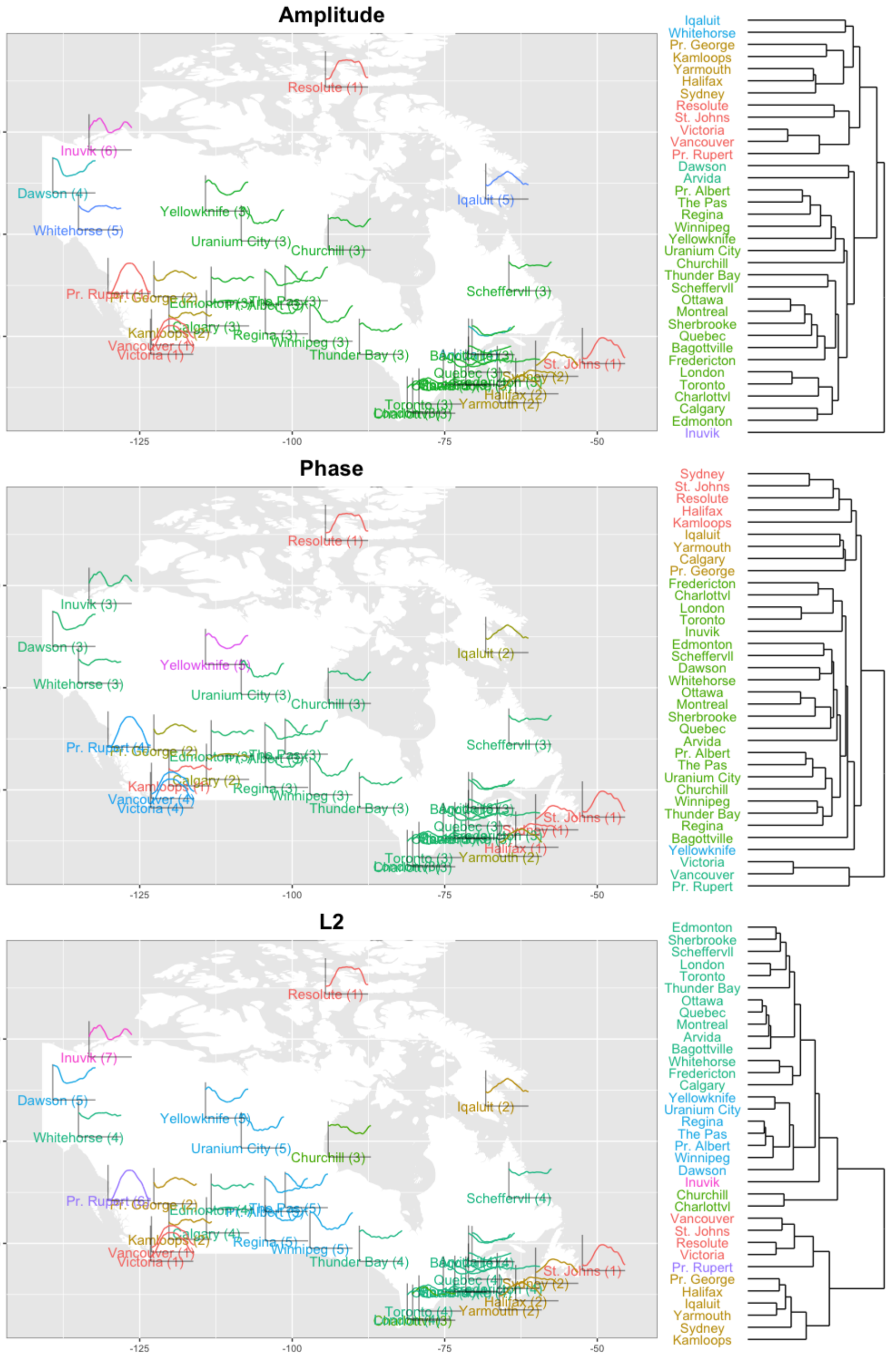

Figure 9: Clustering (average linkage, clusters in different colors) of functional residuals, after adjusting for latitude and longitude effects, obtained from the Canadian weather data when dissimilarity does not account for spatial dependency.
